# A suppressor tRNA-mediated feedforward loop eliminates leaky gene expression in bacteria

**DOI:** 10.1093/nar/gkaa1179

**Published:** 2020-12-08

**Authors:** Joanne M L Ho, Corwin A Miller, Sydney E Parks, Jacob R Mattia, Matthew R Bennett

**Affiliations:** Department of Biosciences, Rice University MS-140, 6100 Main St., Houston, TX 77005, USA; Department of Biosciences, Rice University MS-140, 6100 Main St., Houston, TX 77005, USA; Department of Biosciences, Rice University MS-140, 6100 Main St., Houston, TX 77005, USA; Department of Biosciences, Rice University MS-140, 6100 Main St., Houston, TX 77005, USA; Department of Biosciences, Rice University MS-140, 6100 Main St., Houston, TX 77005, USA; Department of Bioengineering, Rice University MS-140, 6100 Main St. Houston, TX 77005, USA

## Abstract

Ligand-inducible genetic systems are the mainstay of synthetic biology, allowing gene expression to be controlled by the presence of a small molecule. However, ‘leaky’ gene expression in the absence of inducer remains a persistent problem. We developed a leak dampener tool that drastically reduces the leak of inducible genetic systems while retaining signal in *Escherichia coli*. Our system relies on a coherent feedforward loop featuring a suppressor tRNA that enables conditional readthrough of silent non-sense mutations in a regulated gene, and this approach can be applied to any ligand-inducible transcription factor. We demonstrate proof-of-principle of our system with the lactate biosensor LldR and the arabinose biosensor AraC, which displayed a 70-fold and 630-fold change in output after induction of a fluorescence reporter, respectively, without any background subtraction. Application of the tool to an arabinose-inducible mutagenesis plasmid led to a 540-fold change in its output after induction, with leak decreasing to the level of background mutagenesis. This study provides a modular tool for reducing leak and improving the fold-induction within genetic circuits, demonstrated here using two types of biosensors relevant to cancer detection and genetic engineering.

## INTRODUCTION

Ligand-inducible genetic control systems are the bedrock of synthetic biology, rendering the expression of any gene of interest dependent upon the addition of a small molecule inducer. To date, efforts continue to be made both to improve and characterize existing inducible biosensors, as well as to develop novel biosensors that respond to increasingly diverse molecular inducers (1,2). Inducible biosensors can be linked to more complex functions by coupling them to other engineered regulatory elements, such as multi-input transcriptional logic gates (3), STAR elements (4), insulators (5), riboregulators (6) and attenuators (7). Together, these components have enabled the development of diverse cellular operations such as cellular memory (8), oscillations (9), diagnostics (10), triggered drug delivery (11) and complex multilayer genetic programs (12,13). Proper execution of increasingly complex operations, however, requires the individual genetic circuit components to perform robustly and mediate precise gene expression.

‘Leaky’ gene expression, wherein expression of a regulated gene is observed in its uninduced state, remains a persistent problem in synthetic biology and often contributes to the poor performance of genetic circuits. Low levels of leaky expression are notably required for the inducible expression of toxic genes (14), including toxic counterselection markers such as barnase (15) and inducible mutagenesis genes (16). Indeed, many useful ligand-inducible transcription factors used in synthetic biology, including the extensively used arabinose-inducible AraC, exhibit significant measurable leak with toxic genes (16) and sensitive reporters (17).

To address the problem of leaky expression, scientists have previously built AND logic gates that combine transcriptional control systems with a new translational regulatory module (18,19). These studies relied on previously engineered pairs of tRNA and aminoacyl-tRNA synthetases (aaRSs) that are orthogonal to the cellular aminoacylation machinery (15) to incorporate noncanonical amino acids (ncAAs) into genes of interest. By placing individual components of an orthogonal aaRS·tRNA pair under the transcriptional control of different inducers and building a positive feedback loop, scientists have achieved near-zero leak with minimal signal loss (18,19). Drawbacks of this system, however, include its requirements for (i) inserting one or more ncAAs into the coding sequence of regulated proteins, (ii) using multiple chemical inducers and (iii) utilizing a ncAA for which aminoacylation by its corresponding aaRS is inefficient. Thus, we sought to engineer a simpler and more generalizable leak reduction system that combines transcriptional and translational control while using only canonical amino acids.

## MATERIALS AND METHODS

### Preparation of leak dampener module

The gene encoding suppressor tRNA *supP* is an anticodon mutant of *leuX* (EcoCyc EG30053; Genbank NC_000913.3 MG1655 region 4496405–4496489). Mutation of the *leuX* tRNA anticodon from CAA to CUA (BBa_K1499251) enables decoding of amber (TAG) stop codons, which are translated as the amino acid leucine (20). The *supP* gene was synthesized as oligonucleotides (Sigma), PCR assembled, and cloned into a pTech backbone (a generous gift from Prof. Dieter Söll at Yale University), which is optimized for tRNA overexpression. In turn, mutation of the *leuX* tRNA anticodon from CAA to UCA enables decoding of opal (TGA) stop codons, which can then be translated as leucine. LeuRS is able to aminoacylate *supP* (amber-decoding) and *supP_UCA_* (opal-decoding) in addition to all the endogenous leucine tRNA species because of its anticodon-independent substrate recognition mechanism. To enable proper repression of *supP* before induction and inducible overexpression of the mature tRNA, the gene encoding a cis-acting hammerhead ribozyme (21) was cloned preceding *supP* in the pTech backbone. This hammerhead ribozyme had previously been developed to remove undesired appendages from the 5′ terminus of tRNA transcripts, via autocatalytic cleavage just before nucleotide +1 of the tRNA. We deposited a set of plasmids we constructed, along with their maps and sequences, in the public repository Addgene (Supplementary Table S1). Sequences of key plasmids are provided in the Supplementary Data Files.

### Preparation of biosensor and reporter plasmids

DNA encoding the LldR transcription factor (*lldR*) and its promoter sequence Plldr (generous gifts from Prof. Paul Freemont at Imperial College London) were PCR amplified and inserted via Gibson assembly into a pJKR-H backbone (a generous gift from Prof. George Church at Harvard Medical School), which is optimized to maximize the signal-to-noise ratio of transcription factor biosensors regulating expression of a reporter protein, green fluorescence protein. Amber or opal codons were installed at permissive sites (N39O, N135O and Y151O) and leucine codons of green fluorescence protein. These permissive sites were chosen as prior biochemical studies found these positions to be highly tolerant to coding mutations (22–24). To construct the leak dampened mutagenesis plasmid MP6.6TAG, amber codons were inserted after the initiator methionine codon of each of the six mutagenic genes (*dnaQ926*, *dam*, *seqA*, *emrR*, *ugi* and *cda1*) originally present in the mutagenesis plasmid MP6 (a generous gift from Prof. David Liu at Broad Institute). Stop codons in reporter genes were introduced by QuikChange mutagenesis or PCR followed by Gibson assembly. We deposited a set of plasmids we constructed, along with their maps and sequences, in the public repository Addgene (Supplementary Table S1). Sequences of key plasmids are provided in the Supplementary Data Files.

### Fluorescence-based stop codon readthrough assay

Cells (C321.ΔA.exp or *E. coli* Turbo) were transformed with the pJKR-H-derived reporter plasmids and plated on LB agar supplemented with antibiotics. Strain C321.ΔA.exp was a generous gift from Prof. George Church. For each sample, individual colonies were picked in biological triplicates and grown overnight in LB media supplemented with antibiotics, with shaking at 37°C. Cultures were back diluted 1:100 and grown in LB media to early exponential phase (*A*_600_ = 0.1), at which point expression of the feedforward loop was induced with sodium L-lactate (Sigma) serially diluted to a test concentration of 0, 1, 10 or 100 mM, to induce production of the fluorescence reporter. Cells were distributed into a 96-well U-bottom microplate (Falcon) and analyzed using a Tecan Spark multimode microplate reader. Fluorescence intensity (excitation 488 nm, emission 509 nm) and optical density (*A*_600_) were tracked over time for samples with varying numbers of stop codons (1-3,10,15 or 20). Experiments were performed in biological triplicates. Fluorescence intensity values were normalized by cell optical density and no background correction (media or cellular autofluorescence) was performed. Raw values of fluorescence normalized by *A*_600_ were used to compute improvements in fold-change induction, signal retention, leak reduction, and fold-change improvement (Table 1), as well as suppression (readthrough) efficiency and leaky expression (Figures 2–4, Supplementary Figures S2 and S4; Supplementary Tables S2–S6). Suppression efficiency was calculated as the fluorescence/*A*_600_ value of a given sample containing stop codons divided by the fluorescence/*A*_600_ value of the otherwise identical sample that does not contain any stop codons within GFP. For each sample, the average of three biological replicates and the corresponding standard deviation values were calculated. For statistical comparisons between samples, two-sample *t*-tests assuming equal variance were performed using Microsoft Excel; reported *P*-values are two-tailed.

**Table 1.** Performance summary. Showcase examples of leak dampener tool applied to different combinations of two biosensors (either LldR or AraC), two types of reporter genes (GFP or mutagenesis genes), two types of stop codons (amber or opal), different quantities of stop codons within regulated genes (between 1 and 15) across three *E. coli* strains with the relevant genomic information provided (presence of release factor and the number of relevant stop codons in the genome). Codon usage values are based on NC_000913.2 (National Center for Biotechnology Information, 1 September 2011) (37). For each example, fold induction is calculated by dividing measured signal in at the highest concentration of inducer used by signal observed in the absence of inducer. Signal retention is calculated by dividing induced signal of leak dampened samples by the induced signal of the corresponding 0 stop codon control. Leak reduction is calculated by dividing measured signal in the absence of induction of the 0 stop codon control by signal measured from the leak dampened samples in the absence of induction. Fold induction improvement is calculated by dividing the fold induction of the leak dampened samples by the fold induction of the 0 stop codon control. Uncertainty values shown are the standard deviation. For AraC/ S1030 MP6.6TAG samples, standard deviations were calculated across four biological replicates; for all other samples, standard deviations were calculated across three biological replications

TF/strain/reporter	LldR/ C321.ΔA.exp/ GFP.15TAG	LldR/Turbo/ GFP.2TAG	LldR/ Turbo GFP.1TGA	AraC/S1030 GFP.2TAG	AraC/S1030 MP6.6TAG
Release factor (RF)	Deleted RF1	Intact	Intact	Intact	Intact
Codon background	0 amber	321 amber	1232 opal	321 amber	321 amber
Inducer concentration	100 mM lactate	100 mM lactate	100 mM lactate	1 mM Ara	10 mM Ara
Fold induction	68.7±4.3	19.2±1.7	124.5±1.0	634.4±20.7	541.8±317.9
Signal retention (%)	109±4	60±4	30±1	91±0.1	25±19
Leak reduction	3.2±0.3	5.2±0.6	37.0±1.2	1.9±0.1	30.2±27.2
Fold induction improvement	3.5±0.2	3.1±0.3	11.1±0.1	1.7±0.1	7.6±5.2

### Rifampicin resistance mutation rate assay

Rifampicin resistance assays were performed as previously described (16). Briefly, three plasmid combinations (pJH474, pJH474 with MP6.6TAG, or pJH474 with MP6) were separately transformed into *E. coli* strain S1030 (a generous gift from Prof. David Liu). Samples were plated on LB plate supplemented with 20 mM d-glucose and antibiotics, and plates were incubated overnight. Four colonies from each plate were picked and grown overnight in 1 ml LB supplemented with 20 mM d-glucose and antibiotics. Each culture was subsequently back-diluted 100-fold into two separate tubes containing 2 ml of LB with antibiotics, and tubes were grown for 1.5 h at 37°C. One batch of tubes were subsequently induced with 10 mM l-arabinose, while the other batch were given 20 mM d-glucose. Cultures were subsequently grown for 18–24 h at 37°C. Serial dilutions (10^0^–10^−6^) of each sample were plated on LB agar plate supplemented with 20 mM d-glucose and were separately plated on plates containing 20 mM d-glucose and 100 μg/ml rifampicin. Plates were wrapped in foil to protect photosensitive rifampin from light, and plates were incubated overnight at 37°C. Colonies were subsequently counted for each plate.

Mutation rates (Supplementary Table S7), denoted as μ_bp_ (substitutions per base pair of the *E. coli* genome per generation), were calculated using the previously described equation μ_bp_ = *f*/[*R* × ln(*N*/*N*_0_)], where *f* is the frequency of rifampin-resistant mutants (CFUs counted on rifampicin plates divided by CFUs counted on glucose plates for each sample), *R* is the number of unique sites yielding rifampin resistance (77 previously identified sites), *N* is the final population size (10^8^) and *N*_0_ is the population size at which resistance is first observed (∼1.5 × 10^7^ based on prior work) (16).

## RESULTS

### Leak dampener tool contains suppressor tRNA and hammerhead ribozyme

**Figure 1.**
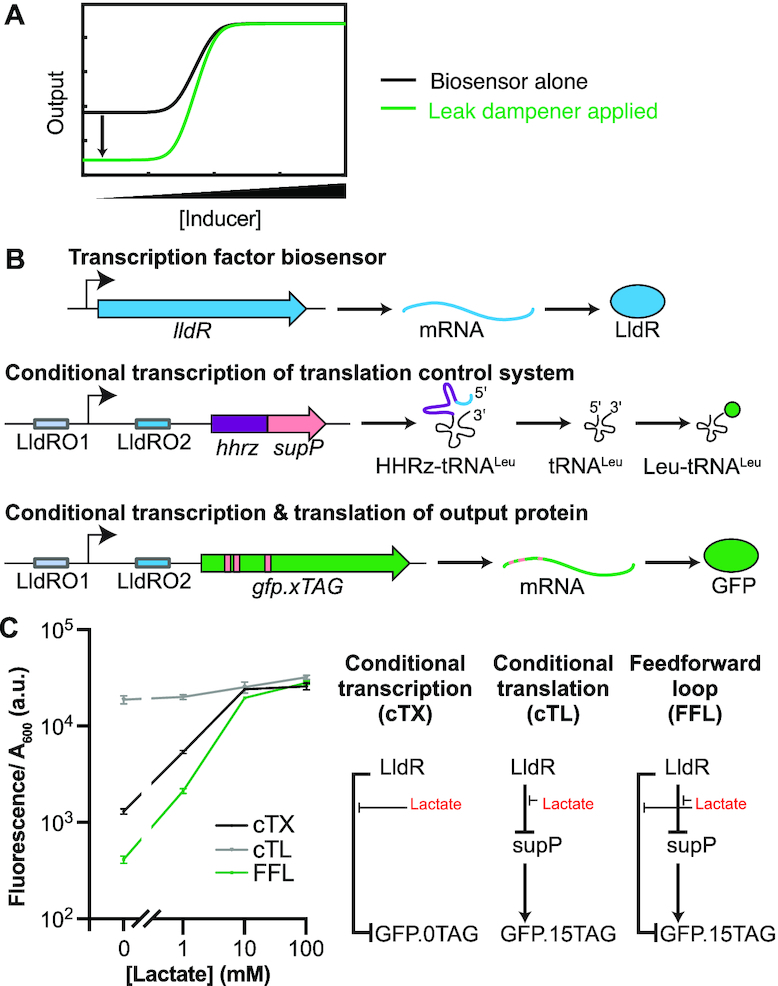
Rationale of the leak dampener tool. (**A**) When applied to an inducible biosensor, our leak dampener is designed to reduce expression levels of the regulated gene (output) in the absence of inducer while maintaining high output in the presence of inducer. (**B**) Application of leak dampener to LldR regulated expression of GFP. In response to lactate, LldR increases transcription levels of both GFP and our self-processing *hhrz-supP* fusion. Increased transcription of *supP* in turn allows more GFP to be expressed by mediating correct translation of leucine codons that have been mutated to TAG. (**C**) LldR can regulate expression of GFP via lactate induction either by conditional transcription (cTX, 20-fold induction) of GFP mRNA or by controlling transcription of *supP*, which in turn mediates conditional translation (cTL, 2-fold induction) of GFP. Our leak dampener system combines both methods of control within a coherent feed-forward loop (FFL, 70-fold induction). Data shows the average across three biological replicates, and error bars show the standard deviation.

In order to decrease the leak while retaining signal (Figure 1A), we designed a Type I coherent feedforward loop in which an inducible transcription factor biosensor controls expression of both a gene of interest and a suppressor tRNA. The suppressor tRNA, in turn, regulates translation of the gene of interest by mediating readthrough of stop codons (Figure 1B). In the absence of an inducer, leaky expression of regulated genes will be greatly reduced because the presence of stop codons will prevent proper translation of leaky mRNA.

Our approach entails the addition of a single component to existing biosensor circuits—specifically, a suppressor tRNA that gets charged by endogenous *E. coli* aaRSs that are already present in the cell. In nature, many nonsense suppressor tRNAs arose through mutation of their anticodons to recognize the stop codons, while maintaining the ability to be recognized and charged by their cognate aaRSs (25–29). Notably, there exists a large variety of suppressor tRNAs with variable suppression efficiencies, with the most efficient reaching as high as 100% readthrough of stop codons depending on the genetic context (25–30). Our circuit can be used to regulate the expression of virtually any protein by ‘silently’ mutating existing codons to stop codons, the correct translation of which will depend upon induced expression of the corresponding suppressor tRNA.

For this work, we chose to use the leucine suppressor tRNA *supP* (20) for our leak dampener module based on three criteria: first, prior studies indicate *supP* is among the most efficient nonsense suppressors in *E. coli* (30), which maximizes signal retention. Second, aminoacylation of leucine tRNAs by their corresponding aaRS, leucyl-tRNA synthetase (LeuRS), takes place via an anticodon-independent recognition mechanism (31). As a result, aminoacylation efficiency remains high, regardless of the anticodon sequence of the leucine suppressor tRNA. Consequently, although *supP* is naturally an amber (TAG) suppressor, it can be readily mutated to produce an opal (TGA) or ochre (TAA) suppressor (30). Third, since leucine is the most commonly occurring amino acid encoded in the *E. coli* genome (32), genes of interest are likely to contain numerous leucine residues that can be silently mutated to stop codons to link their translation to *supP*. This approach not only allows a gene to be regulated by *supP* without altering its amino acid sequence, but also enables *supP* regulation to be optimized by tuning the number of stop codon substitutions made in a given gene (32).

For our first proof-of-principle test, we chose the lactate-responsive repressor LldR as a showcase because of its potential importance in biomedicine which remains stymied by its poor performance. Previous studies have established lactate as a biomarker for cancer in humans, as lactate is found at elevated levels in tumor microenvironments in a phenomenon termed the Warburg effect (33). LldR thus has the potential to serve as a cancer biosensor, yet regulation by LldR is notably leaky and produces a poor fold change in output after induction; in a recent report, the highest fold change in output reported was 8-fold (34).

**Figure 2.**
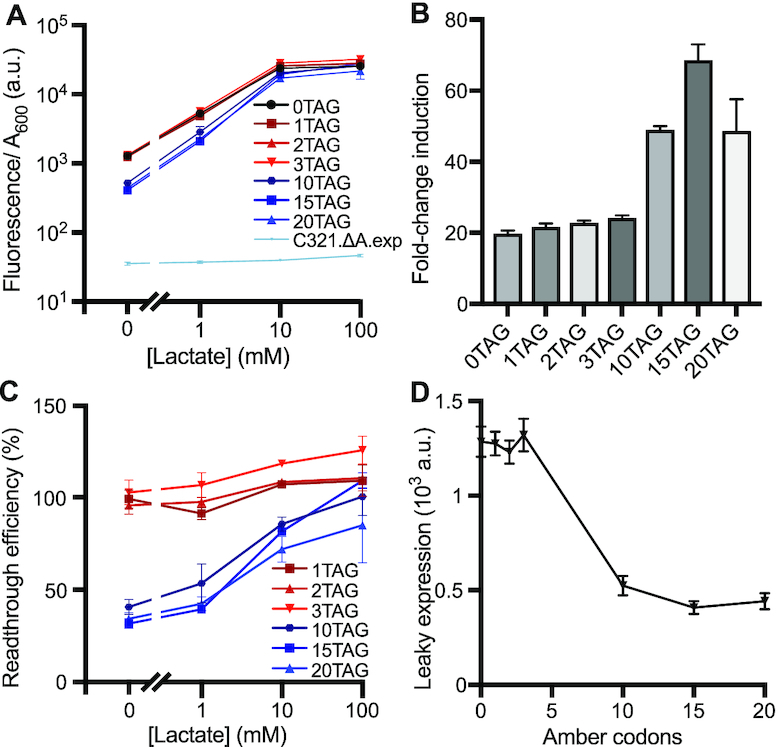
Leak dampener tool applied to LldR induction using amber codons in *E. coli* C321.ΔA.exp. (**A**) Fluorescent response curves of GFP variants containing variable numbers of amber (TAG) codons are shown following lactate induction of our leak dampener system. Use of the ‘amberless’ *E. coli* strain C321.ΔA.exp allows large number of amber codons to be efficiently suppressed by *supP* with high signal retention (85–126%). (**B**) Fold-change induction is shown for each GFP variant, calculated by dividing fluorescence/*A*_600_ in the presence of 100 mM lactate by the observed fluorescence/*A*_600_ in the absence of lactate. The greatest improvement (3.5-fold) in fold induction is observed for GFP containing 15 amber codons. (**C**) Readthrough (amber suppression) efficiency is shown for each GFP variant at each lactate concentration, calculated by dividing fluorescence/*A*_600_ signal observed for each GFP variant by the signal observed for GFP containing 0 stop codons. Read-through efficiency increases with inducer concentration, mediated by increased expression of *supP*. (**D**) Leaky gene expression, defined as fluorescence/*A*_600_ signal observed in the absence of lactate inducer, is shown as a function of the number of amber codons present in regulated GFP variants. Leak is observed to decrease as the number of amber codons increases, with 2.5–3.2-fold leak reduction achieved with 10 or more amber codons. Data shows the average across three biological replicates, and error bars show the standard deviation.

We first improved LldR with a conventional approach by cloning codon-optimized LldR into a pJKR-H backbone, a high copy number plasmid with a strong promoter and ribosome binding sites that has previously been optimized to maximize the signal-to-noise ratios of a variety of transcription factor biosensors (35). This plasmid, termed pJH625, produced a 20-fold change in GFP output after induction (Figure 2), as determined by a fluorescence plate reader assay performed as previously described (36) without any background correction performed.

Initially, using LldR to control the expression of *supP* proved challenging due to constraints on the presence and positioning of its operator sequences. Upon application of our leak dampener tool to pJH-LldR, we observed that in order to repress *supP* expression before induction, both LldRO1 and LldRO2 sites had to be included, and LldRO2 had to be placed after the transcription initiation site (Supplementary Figure S1A). This promoter architecture resulted in an undesired extension on 5′ end of the tRNA, abolishing its suppressor function. Thus, in consideration of future promoter architectures that may similarly contain operator sequences after the transcription initiation site and in order to eliminate the need for extensive promoter engineering prior to applying our tool, we introduced a hammerhead ribozyme (HHRz) (21) between the LldO2 operator site and *supP*. Following its transcription this ribozyme cleaves the base at its 3′ end, mediating correct processing of the downstream tRNA and yielding functional *supP* (Supplementary Figure S1B and C). In contrast to *supP*, biological activity of HHRz is unaffected by extensions to its 5′ end. Combining HHRz with *supP* thus produces a modular self-processing tool, allowing expression of mature *supP* to be controlled by any desired promoter or operator sequence regardless of variations at or after the transcription start site (21).

### Leak dampener reduces leaky output of lactate biosensor LldR

We applied our leak dampener module to the lactate-inducible LldR repressor and tested it in two different *E. coli* strains. The first, C321.ΔA.exp, is a recoded *E. coli* strain with innate amber termination abolished through the removal of amber codons from the genome and the deletion of release factor 1; as such, this strain enables efficient readthrough of numerous amber codons and is extensively used in genetic code expansion studies (37,38). In contrast, *E. coli* NEB Turbo is a standard *E. coli* strain and contains release factor 1. Within the distinct suppression environments of these two strains, we subsequently tested readthrough of GFP mutants containing variable numbers of amber codons to evaluate the effects of this parameter on leak reduction and fold-induction.

We first tested our leak dampener circuit in strain C321.ΔA.exp. Upon testing pJH-LldR paired with GFP variants containing between 0 and 20 amber codons, we observed significant (1.0–3.2-fold) leak reduction. Comparing leaky expression for GFP variants with 3 versus 10 amber codons, the difference is statistically significant (*P* = 2.8 × 10^−9^); in contrast, comparing the leak of 10 versus 15 amber codon variants, the difference is not significant (*P* = 0.23). These results indicate that beyond 10 amber codons, additional amber codons confer diminishing returns towards leak reduction. We also observed 1.1–3.5-fold improved fold-induction and excellent (85–126%) signal retention for all GFP variants containing varying numbers of amber codons (1-3,10,15, and 20), relative to the 0 amber codon control at all lactate concentrations tested (Figure 2A). In this context, we observed the best performance for GFP containing 15 amber codons, which compared to the 0 amber codon control exhibited 3.2-fold reduced leak in the absence of lactate and 3.5-fold increased fold-induction in the presence of 100 mM lactate (Table 1). Notably, we observed negligible signal loss for each amber-containing GFP variant tested in this strain, with GFP.15TAG exhibiting 109% signal compared to the 0 stop codon control in the presence of 100 mM lactate. Comparing induced GFP expression at 100 mM lactate for our 0 versus 20 amber codon variants, the difference was not statistically significant (*P* = 0.12). With 10–20 amber codons, fold-change induction was improved by 2.5–3.5-fold (Figure 2B), readthrough efficiency rose from 32–41% through 85–109% with increasing lactate concentration (Figure 2C), and 2.5–3.2-fold leak reduction was achieved (Figure 2D). Despite the considerable leak reduction that we achieved using this strain, we were unable to completely eliminate leak; all of our amber-containing variants exhibited higher leak compared to the negative control (Figure 2A). In light of these results, when using amber codons in strain C321.ΔA.exp our 15 amber codon construct appears to be the most effective for retaining signal and maximizing fold induction.

**Figure 3.**
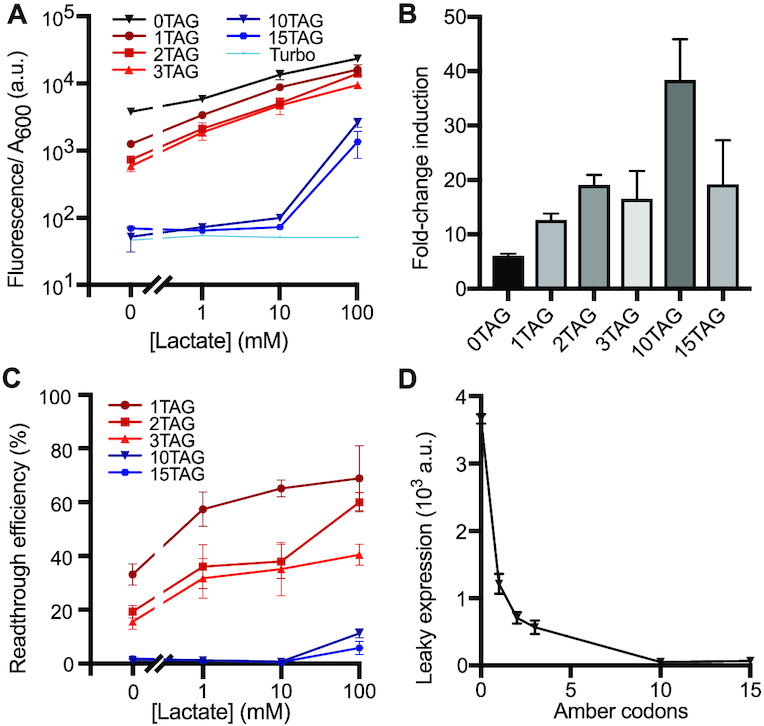
Leak dampener tool applied to LldR induction using amber codons in *E. coli* NEB Turbo. (**A**) Fluorescent response curves of GFP variants containing variable numbers of amber (TAG) codons are shown following lactate induction of our leak dampener system. Compared to strain C321.ΔA.exp, use of a standard *E. coli* cloning strain (NEB Turbo) results in greater leak reduction (3–85-fold) for each amber codon present in GFP, but moderate signal retention (6–69%). (**B**) Fold-change induction is shown for each GFP variant, calculated by dividing fluorescence/*A*_600_ in the presence of 100 mM lactate by the observed fluorescence/*A*_600_ in the absence of lactate. The greatest improvement (9.5-fold) in fold induction is observed for GFP containing 10 amber codons. (**C**) Readthrough (amber suppression) efficiency is shown for each GFP variant at each lactate concentration, calculated by dividing fluorescence/*A*_600_ signal observed for each GFP variant by the signal observed for GFP containing 0 stop codons. Read-through efficiency increases with inducer concentration (mediated by increased expression of *supP)* but decreases for GFP variants containing greater numbers of amber codons due to competition with release factor 1. (**D**) Leaky gene expression, defined as fluorescence/*A*_600_ signal observed in the absence of lactate inducer, is shown as a function of the number of amber codons present in regulated GFP variants. Leak is observed to decrease as the number of amber codons increases, with significant leak reduction (3-fold) achieved with a single amber codon and 85-fold leak reduction achieved with 10 amber codons. Data shows the average across three biological replicates, and error bars show the standard deviation.

We next tested our leak dampener circuit in *E. coli* strain NEB Turbo, wherein release factor 1 competes with *supP* at amber codons. Compared to strain C321.ΔA.exp, the Turbo strain exhibited much higher leak reduction (3–85-fold); however, this improved leak reduction was also accompanied by a larger loss of signal, with 6–69% signal retention at 100 mM lactate relative to the 0 amber codon control (Figure 3A). In NEB Turbo, substantial (3–7-fold) leak reduction and high (40–69%) signal retention was observed for GFP variants containing 1, 2 and 3 amber codons relative to the 0 amber codon control (Figure 3A). For 1, 2, and 3 amber codons, fold-change induction was improved by 2.1–3.1-fold (Figure 3B) and high readthrough efficiency (40–69% at 100 mM lactate) was observed (Figure 3C). A substantial 3-fold leak reduction was easily achieved with just one amber codon (Figure 3D). In NEB Turbo, we observed the best performance using GFP containing two amber codons, finding that, compared to the 0 amber codon control, leaky gene expression was reduced by 5.2-fold in the absence of lactate, fold-induction was increased by 3.1-fold in the presence of 100 mM lactate, and signal retention was 60% in the presence of 100 mM lactate (Table 1). Comparing leaky expression between our 10 and 15 amber codon variants, the difference is not statistically significant (*P* = 0.23), indicating maximal leak reduction is achieved in this strain with 10 amber codons. Notably, any given variant (e.g. GFP.10TAG) always exhibits a greater reduction of leak (85- versus 2.5-fold) and lower retention of signal (11% versus 100%) when tested in NEB Turbo rather than C321.ΔA.exp—an observation consistent with the effects of release factor 1 that is present to terminate stalled translation of amber codons in NEB Turbo but not C321.ΔA.exp. In light of these results, when using amber codons in strain NEB Turbo our 1–3 amber codon constructs is the most effective for retaining signal, whereas our 10 amber codon construct is best for minimizing leak and maximizing fold induction.

In conclusion, LldR can regulate expression of GFP via lactate induction either by conditional transcription (cTX, 20-fold induction) of GFP mRNA or by controlling transcription of supP, which in turn mediates conditional translation (cTL, 2-fold induction) of GFP.15TAG (Figures 1C, 2 and Supplementary Figure S2; Supplementary Tables S2 and S5). By combining both methods of control within a coherent feed-forward loop (FFL, 70-fold induction), our leak dampener system provides a 3.5-fold improvement in the fold induction.

### Leak dampener operates with opal (TGA) stop codons

**Figure 4.**
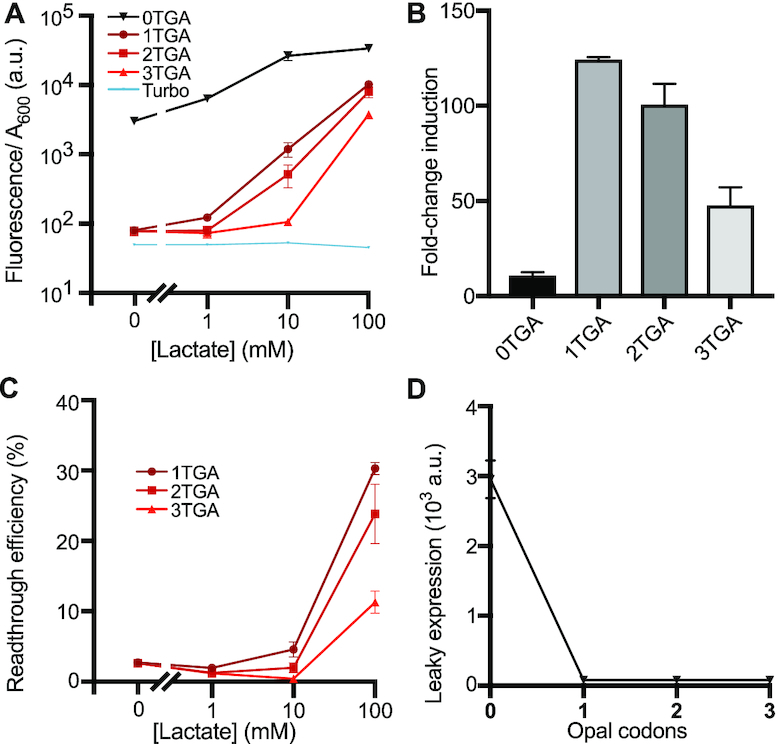
Leak dampener tool applied to LldR induction using opal codons in *E. coli* NEB Turbo. (**A**) Fluorescent response curves of GFP variants containing variable numbers of opal (TGA) codons are shown following lactate induction of our leak dampener system. Compared to use of amber codons in strain NEB Turbo, use of opal codons results in greater leak reduction (37–39-fold) for each stop codon present in GFP and moderate signal retention (11–30%). (**B**) Fold-change induction is shown for each GFP variant, calculated by dividing fluorescence/*A*_600_ in the presence of 100 mM lactate by the observed fluorescence/*A*_600_ in the absence of lactate. The greatest improvement (11-fold) in fold induction is observed for GFP containing a single opal codon. (**C**) Readthrough (opal suppression) efficiency is shown for each GFP variant at each lactate concentration, calculated by dividing fluorescence/*A*_600_ signal observed for each GFP variant by the signal observed for GFP containing 0 stop codons. Read-through efficiency increases with inducer concentration (mediated by increased expression of *supP_UCA_*) but decreases for GFP variants containing greater numbers of opal codons due to competition with release factor 2. (**D**) Leaky gene expression, defined as fluorescence/*A*_600_ signal observed in the absence of lactate inducer, is shown as a function of the number of opal codons present in regulated GFP variants. Leak reduction is maximized (11-fold) following the insertion of a single opal codon in GFP. Data shows the average across three biological replicates, and error bars show the standard deviation.

We next mutated the anticodon of *supP* from CUA to UCA, producing an opal suppressor tRNA variant termed *supP_UCA_*. We also prepared mutant GFP variants containing 1–3 opal (TGA) codons, and next tested the efficacy of our lactate-responsive opal leak dampener circuit in strain NEB Turbo. We observed significant (37–39-fold) leak reduction and moderate (11–30%) signal retention for 1, 2 and 3 opal codons (Figure 4A). Fold-change induction was drastically improved by 4–11-fold (Figure 4B) and moderate readthrough efficiency (11–30% at 100 mM lactate) was observed in all cases (Figure 4C). Using opal codons in *E. coli* Turbo, we observed the best leak reduction performance with just one opal codon (Figure 4D). Compared to the 0 opal codon control, the GFP variant containing a single opal codon reduced leaky gene expression by 37-fold in the absence of lactate, and increased fold-induction by 11.1-fold in the presence of 100 mM lactate (Table 1). Comparing leaky expression of our 1 and 2 opal codon variants, the difference is not statistically significant (*P* = 0.73), indicating maximal leak reduction is achieved in this strain with a single opal codon. Notably, GFP.1TGA exhibited 30% as much signal as the 0 stop codon control in the presence of 100 mM lactate. These results demonstrate that the leak dampening effect mediated by opal codons mediates a greater reduction of leak but also a greater loss of signal compared to an equivalent number of amber codons. These findings are consistent with prior reports showing that amber suppression is generally more efficient than opal suppression in *E. coli* (30). In light of these results, when using opal codons in strain NEB Turbo our 1 opal codon constructs is the most effective for retaining signal and maximizing fold-induction, while our 3 opal codon construct is best for minimizing leak.

### Growth of NEB Turbo encoding the leak dampener circuits

Since NEB Turbo contains 321 instances of amber codons, we were curious about the impact of expressing the suppressor tRNA on cellular fitness. At high lactate concentrations, amber suppression by the suppressor tRNA will elongate those 321 endogenous genes. To determine whether expression of the leak dampener circuit causes any growth defect in NEB Turbo, we compared the growth for the best performing GFP variants, using amber and opal codons for different levels of induction (Supplementary Figure S3 and Supplementary Tables S2–S6). The jagged shape of the growth curves at 16 h (Supplementary Figure S3) reflects the tendency of cells to sediment when grown overnight in a plate shaker. For all our fluorescence readthrough experiments, cells are resuspended at the final timepoint (18 h post-induction) before taking the measurements (Supplementary Tables S2–S6; Figures 2–4, Supplementary Figures S2 and S4). Of note, regardless of whether it encodes the leak dampener circuit, NEB Turbo always grows to a higher density at higher lactate concentrations (Supplementary Figure S3a versus Supplementary Figure S3b–d; Supplementary Tables S3–S4) since lactate is a carbon source.

While NEB Turbo encoding the amber codon leak dampener circuit grows at a slower rate than NEB Turbo alone, both of them reach the same total growth and share the same final *A*_600_ (Supplementary Figure S3). With NEB Turbo encoding the opal codon leak dampener circuit, the growth rate and total growth are identical to that of NEB Turbo alone (Supplementary Figure S3). Interestingly, while the final *A*_600_ is identical for both types of leak dampener circuits, the deleterious effect of the amber suppressor tRNA on growth rate is greater than that of the opal suppressor tRNA. Prior studies have found that amber and opal suppressors have little to no effect on cell fitness (30). Our growth data indicates that fluorescence/*A*_600_ is not artificially inflated by depressed *A*_600_ values, and it is a fair and accurate metric to evaluate functionality of our leak dampener.

### Leak dampener eliminates leak of an arabinose-inducible mutagenesis plasmid

**Figure 5.**
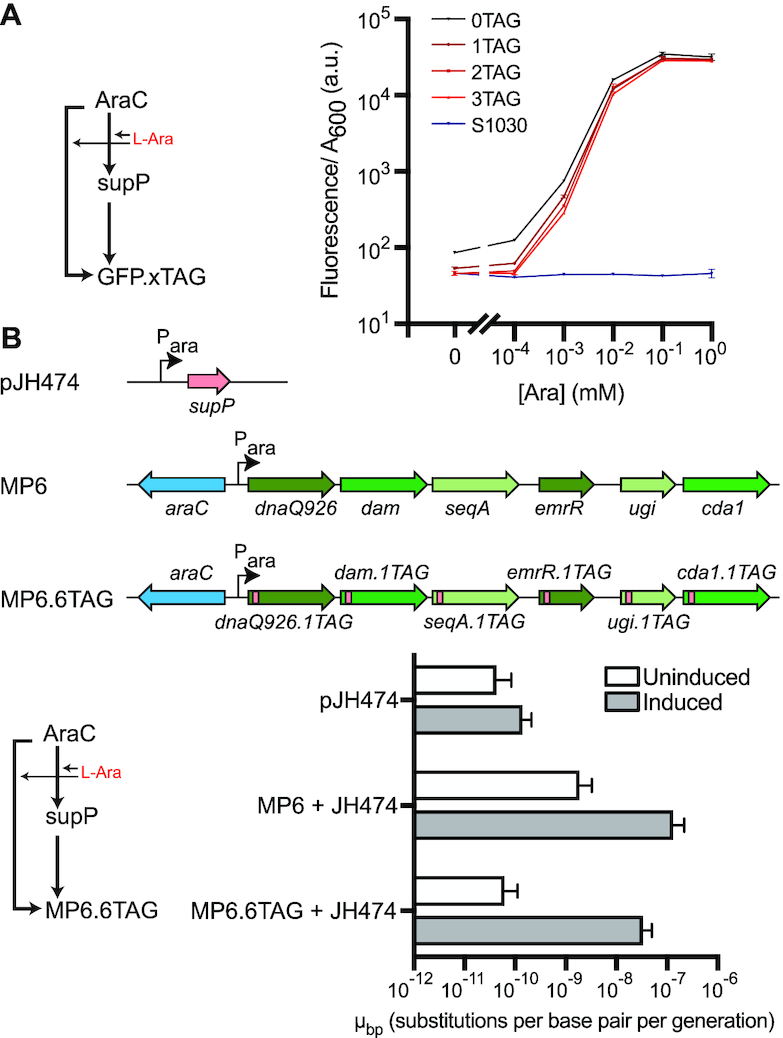
Application of amber leak dampener tool to arabinose induction of reporter genes in *E. coli* S1030 containing a normal codon background. (**A**) Fluorescent response curves of GFP variants containing variable numbers of amber (TAG) codons are shown following arabinose induction of our leak dampener tool applied to AraC, which is considered the industry standard for low leak inducible gene expression. With a fluorescence reporter, application of the leak dampener to AraC results in high signal retention (88–94%) and provides measurable leak reduction (1.6–1.9-fold). Fluorescence data shows the average across two biological replicates, and error bars show the standard deviation. See, Supplementary Table S4 for the complete data. (**B**) Substantial leak in AraC-controlled expression is observed when the regulated genes are toxic, e.g. in the previously described mutagenesis plasmid MP6 that contains six mutagenic genes (*dnaQ926*, *dam*, *seqA*, *emrR*, *ugi*, and *cda1*) under the control of AraC. To generate MP6.6TAG, we inserted an amber stop codon immediately after the start codon in each of the six genes in MP6. To complete the leak dampener circuit, we also cloned *supP* under the control of AraC in a separate vector (pJH474). *E. coli* strain S1030 cells were transformed with either pJH474, MP6.6TAG with pJH474, or MP6 with pJH474. Mutagenesis rates (μ_bp_) were measured following either induction with 10 mM arabinose or repression with 20 mM glucose using a rifampicin resistance based assay (see Materials and Methods). Application of the leak dampener tool to MP6 drastically reduces leaky mutagenesis (30-fold) to background levels, while retaining (25%) a high post-induction mutagenesis rate. Fold change in output after induction rises from 71-fold to 542-fold, which constitutes a 7.6-fold improvement in fold induction. Mutagenesis data shows the average across four biological replicates, and error bars show the standard deviation.

We next applied our leak dampener tool to the arabinose-inducible activator AraC, a biosensor frequently used for low leak expression in biomedical and biotechnological research (14). We began by cloning a new leak dampener circuit in which AraC regulated expression of *supP* as well as GFP variants containing different numbers of amber codons (Figure 5A). Using this system in fluorescence reporter assays (Figure 5A), we observed a 1.6–1.9-fold reduction of leaky expression and a 1.5–1.7-fold improvement in fold-change induction of GFP variants containing 1–3 amber codons compared to the 0 amber codon control. Comparing leaky expression of GFP containing two amber codons to the signal from empty cells, the difference is not statistically significant (*P* = 0.83), indicating that leak was completely eliminated in this sample. Notably, the difference in expression levels induced with 1 mM arabinose between our 0 and 3 amber codon variants was not statistically significant (*P* = 0.22), indicating negligible loss of signal. This improvement in leak reduction is remarkable given that AraC is already a preferred system within the field for experiments requiring low levels of leak.

To demonstrate complete elimination of leak using a more sensitive reporter and also apply our improved AraC biosensor in a biotechnologically relevant setting, we precisely measured the extent of leak elimination using inducible expression of mutagenic genes, utilizing a system that was previously developed to introduce mutations during phage-assisted evolution experiments (16,39,40). We began with mutagenesis plasmid MP6, a vector composed of six mutagenic genes (*dnaQ926*, *dam*, *seqA*, *emrR*, *ugi* and *cda1*) whose expression is regulated by AraC (16). In the absence of induction, MP6 has previously been noted to exhibit substantial leak (16), which is problematic because the elevated basal mutation rate in the uninduced host cell reduces fitness.

To reduce the leak of MP6, we inserted an amber codon immediately after the initiator methionine codon in each of the six mutagenic genes, thereby making plasmid MP6.6TAG (Figure 5B). We then completed our leak dampener circuit by cloning the amber suppressor *supP* under the control of AraC on a separate plasmid (termed pJH474). *E. coli* S1030 is a standard *E. coli* strain containing release factor 1 and was developed to be used as the host strain in phage-assisted evolution experiments. We subsequently used a previously described rifampicin resistance assay (see Materials and Methods) to measure the mutation rates of MP6, our leak-dampened system, as well as a negative control in both the presence and absence of arabinose induction (Figure 5B and Supplementary Table S7). In the absence of arabinose induction, cells containing our leak-dampened system exhibited 30-fold less leak compared to MP6. Leaky mutagenesis, in units of substitutions per base pair per generation (μ_bp_), decreased from 1.91 × 10^−9^ μ_bp_ for MP6, to 6.32 × 10^−11^ μ_bp_ for MP6.6TAG, with our uninduced leak dampened system exhibiting a mutation rate that was not significantly different (*P* = 0.22) to that of the negative control (4.41 × 10^−11^ μ_bp_) (Figure 5b and Supplementary Table S7). When induced with 10 mM arabinose, our leak dampened system exhibited a 7.6-fold improvement in fold induction compared to MP6, while retaining 25% signal (Table 1).

## DISCUSSION

This study demonstrates the first use of canonical amino acid suppressor tRNAs as a control system, with our leak dampener joining a growing body of tools for controlling gene expression (3–7). Our leak dampener is a highly modular genetic component, which is compatible with diverse biosensor inputs (AraC and LldR) and genetic outputs (GFP and six mutagenic genes). Its modularity is enabled both by the inclusion of HHRz, which allows expression of the leak dampener to be controlled by any promoter or operator sequence, and also by the common frequency of leucine codons, which allow *supP* to control translation of a wide variety of genes through conditional readthrough of silent leucine-to-stop codon mutations. Taken together, these features allow our leak dampener to be incorporated into a wide variety of inducible genetic systems and biosensors to reduce leaky expression and improve the fold-induction.

Akin to prior studies illustrating that gene expression can be tuned by altering ribosome binding sites or promoter sequences, we show here that nonsense suppressor regulation can similarly be tuned by altering the number of stop codons within regulated genes. Using other systems that rely on changes to the protein coding sequence (18,19), introducing multiple stop codons would likely have a significant effect on the biological activity of many regulated genes. As our approach utilizes conditionally silent mutations, we are able to avoid this complication. Using LldR regulation of GFP as a test case, we demonstrate tuning of stop codons across three separate contexts. Using suppression of amber codons within the ‘amberless’ *E. coli* strain C321.ΔA.exp, we observed the lowest amount of signal loss, finding that maximal fold induction is attained when suppressing a relatively large number of amber codons (∼15) in the target gene. Our results evaluating suppression of amber codons in strain NEB Turbo demonstrate that our amber leak dampener is effective in a more standard cloning strain, and further show that the optimal number of suppressed amber codons is significantly lower (2-3).

Using our opal suppression leak dampener, we found that insertion of a single codon produced the largest fold-change induction, however a large degree of signal loss was also observed. These findings are consistent with prior studies which have shown opal suppressors to be less efficient than amber suppressors, owing to numerous reasons that remain only partially understood (30). Notably, our opal and amber based leak dampener systems can be combined within the same cell for future applications, used either within separate genetic circuits or together regulating the same gene for greater leak dampening effect. These results demonstrate the versatility of our leak dampener, and can be used to control the dose response curve of any genetic circuit that relies on a protein reporter.

For lactate induction experiments in strain NEB Turbo, we observed that larger amounts of either amber or opal codons (shown in Figures 3A and 4B, respectively) are capable of reducing leak to background levels. However, leak is reduced but not completely eliminated in the recoded strain C321.ΔA.exp (Figure 2A), likely because the absence of release factor 1 prevents translation termination in the absence of suppression. This attribute of strain C321.ΔA.exp likely also accounts for the excellent signal retention observed in this strain. Based on these findings, strain C321.ΔA.exp provides an optimal strain for future experiments that prioritize signal retention, whereas experiments that prioritize leak reduction would be better advised to use a strain containing release factor 1 (such as NEB Turbo).

Among other design considerations, future users of our leak dampener tool may benefit from considering the potential effects of truncated protein produced from regulated genes. In the absence of suppressor tRNA induction, leaky expression of genes containing stop codons are expected to produce truncated protein as the majority translation product, with some full-length mutant protein expressed as the minority product due to the effects of missense translation (41). While these gene products are likely to be expressed at a low level, certain truncated proteins may have a toxic effect on cells due to poor solubility and/or aggregation. While we did not observe such an effect in this work, should toxicity be observed in future experiments, it may be resolved by relocating suppression sites to less problematic regions of the reporter protein.

Given that the microenvironment of normal tissues contains 1.5–3 mM lactate while that of cancer tissues contains 10–30 mM lactate (33,42), lactate is an excellent cancer biomarker and ideal inducer of anti-cancer therapeutics. Despite the importance of lactate as a cancer biomarker, potential biotechnology applications of lactate biosensor LldR are complicated by the poor fold-induction and high leak of this repressor. Application of our leak dampener to LldR greatly reduced leak while increasing fold induction. Here, we show that through the use of amber codons applied to LldR, fold-change induction rises from 20-fold to as high as 69-fold in C321.ΔA.exp (Figure 2B) and from 6-fold to as high as 38-fold in NEB Turbo (Figure 3B). Through the use of opal codons applied to LldR, fold-change induction rises from 11-fold to as high as 124-fold in NEB Turbo (Figure 4B). In contrast, prior reports show 8-fold induction for an optimized circuit, though it should be noted that here we used a higher lactate concentration (100 mM) compared to what was used in prior work (10 mM) (34). Notably, many of our leak-dampened circuits showed a strong response at lactate concentrations below 30 mM, including samples tested in NEB Turbo containing 1–3 amber codons (Figure 3A) or 1–2 opal codons (Figure 4A), as well as all samples tested in C321.ΔA.exp containing 1–20 amber codons (Figure 2A). The improved performance of LldR shown here opens the door to future application in human systems, both as a cancer diagnostic tool and also as a bacterial drug delivery system. With the advent of microbial living therapeutics, the need for cancer-responsive biosensors such as the lactate sensing repressor LldR is evident (34,43). The ability of bacteria to localize at tumors and release drugs in response to tumor microenvironments provides an exciting frontier in cancer therapeutics, however work remains to be done to ensure both safety and efficacy (43).

Within the field, AraC is often viewed as the ‘go to’ standard for low leak inducible gene expression, however leaky expression is still observed when regulating toxic or otherwise sensitive genes (14,16). Mutagenesis plasmid MP6 provides one such system, wherein AraC regulates expression of six toxic mutagenic genes and significant leaky expression has been previously observed (16). After applying our amber suppression leak dampener to this system, we were able to completely abolish all detectable leak, observing that mutation rates reached the level of our background control in the absence of induction. Similarly, when our leak-dampened AraC system was applied to a GFP reporter, we observed complete leak elimination with the use of three amber codons (Figure 5A). While the application of our leak dampener did lead to some signal loss due to the presence of release factor 1, our leak-dampened mutagenesis plasmid exhibited a 7.6-fold improvement in overall fold induction (see Table 1). Expression of toxic genes in *E. coli* is of interest for many purposes, including applications in genetic engineering (16,44) and protein purification (45). Our findings indicate that our leak dampener system is capable of completely ameliorating leaky expression for highly sensitive or toxic systems while still retaining a strong inducible response. In future work, we expect our tool to work well for newly discovered and unmodified biosensors that are of importance to synthetic biology applications, including those recently mined from genomes (1), thereby accelerating studies of such inducible transcription factor biosensors. Also, our leak dampener tool can be used in conjunction with extant control elements and genetic signal amplifiers (46) to improve the performance of complex genetic programs and biosensors. While methods for developing new biosensors are rapidly advancing, biosensors designed to recognize novel ligands often exhibit poor fold-induction (47–49). Genetic amplifiers, comprised of multiple genetic activators interconnected in a synthetic gene cascade, provide a modular approach for increasing a small signal to a large measurable output (46,50,51). However, such systems also amplify leaky expression, an issue for which few prior strategies are able to address. Our leak dampener tool could thus be coupled with genetic amplifiers to further improve the fold-induction of a large variety of inducible biosensors (47–49,52) and complex genetic circuits (12,13) while also reducing leak. Combining these tools will also enable precise control over the dose response curves of key biosensors, since a primary goal in applied synthetic biology is to engineer suitable therapeutic responses at biomedically relevant concentrations of a metabolite. This study will enable the future development of an effective synthetic bacterial cancer therapeutic that provides zero therapeutic output in normal tissue (1–3 mM lactate), and tunable high therapeutic output in tumor microenvironments (10–25 mM lactate) (53).

## Supplementary Material

gkaa1179_Supplemental_File
