# Supplementary material for: A suppressor tRNA-mediated feedforward loop eliminates leaky gene expression in bacteria
Source: Nucleic Acids Res. 2020 Dec 8;49(5):e25. doi: 10.1093/nar/gkaa1179 (PMC7969014; doi:10.1093/nar/gkaa1179)
Supplement: gkaa1179_Supplemental_File [file gkaa1179_supplemental_file.docx]

**Supplementary Information for:**

**A suppressor tRNA-mediated feedforward loop eliminates leaky gene expression in bacteria**

Joanne M. L. Ho^1,^*^,†^, Corwin A. Miller^1,^*, Sydney E. Parks^1^, Jacob R. Mattia^1^, and Matthew R. Bennett^1, 2,†^

^1^ Department of Biosciences, Rice University MS-140, 6100 Main St., Houston, TX 77005, USA

^2^ Department of Bioengineering, Rice University MS-140, 6100 Main St. Houston, TX 77005, USA

* These authors contributed equally.

† Correspondence: joanne.ho@rice.edu, matthew.bennett@rice.edu

**Table of Contents**

1. **Supplementary Data 2–4**
2. **Supplementary Figures 5–8**
3. **Supplementary Tables 9–15**

**I. Supplementary Data**

**Sequence of pJH474.Kan**

ATGAGCCATATTCAACGGGAAACGTCTTGCTCTAGGCCGCGATTAAATTCCAACATGGATGCTGATTTATATGGGTATAAATGGGCTCGCGATAATGTCGGGCAATCAGGTGCGACAATCTATCGATTGTATGGGAAGCCCGATGCGCCAGAGTTGTTTCTGAAACATGGCAAAGGTAGCGTTGCCAATGATGTTACAGATGAGATGGTCAGACTAAACTGGCTGACGGAATTTATGCCTCTTCCGACCATCAAGCATTTTATCCGTACTCCTGATGATGCATGGTTACTCACCACTGCGATCCCCGGGAAAACAGCATTCCAGGTATTAGAAGAATATCCTGATTCAGGTGAAAATATTGTTGATGCGCTGGCAGTGTTCCTGCGCCGGTTGCATTCGATTCCTGTTTGTAATTGTCCTTTTAACAGCGATCGCGTATTTCGTCTCGCTCAGGCGCAATCACGAATGAATAACGGTTTGGTTGATGCGAGTGATTTTGATGACGAGCGTAATGGCTGGCCTGTTGAACAAGTCTGGAAAGAAATGCATAAACTTTTGCCATTCTCACCGGATTCAGTCGTCACTCATGGTGATTTCTCACTTGATAACCTTATTTTTGACGAGGGGAAATTAATAGGTTGTATTGATGTTGGACGAGTCGGAATCGCAGACCGATACCAGGATCTTGCCATCCTATGGAACTGCCTCGGTGAGTTTTCTCCTTCATTACAGAAACGGCTTTTTCAAAAATATGGTATTGATAATCCTGATATGAATAAATTGCAGTTTCATTTGATGCTCGATGAGTTTTTCTAATTTTTTTAAGGCAGTTATTGGTGCCCTTAAACGCCTGGTGCTACGCCTGAATAAGTGATAATAAGCGGATGAATGGCAGAAATTCGAAAGCAAATTCGACCCGGGCGGCCGCAACCCAGCGTTCGATGCTTCTTTGAGCGAACGATCAAAAATAAGTGCCTTCCCATCGAAACCAATTGTCCATATTGCATCAGACATTGCCGTCACTGCGTCTTTTACTGGCTCTTCTCGCTAACCAAACCGGTAACCCCGCTTATTAAAAGCATTCTGTAACAAAGCGGGACCAAAGCCATGACAAAAACGCGTAACAAAAGTGTCTATAATCACGGCAGAAAAGTCCACATTGATTATTTGCACGGCGTCACACTTTGCTATGCCATAGCATTTTTATCCATAAGATTAGCGGATCCTACCTGACGCTTTTTATCGCAACTCTCTACTGTTTCTCCATGCCGAAGTGGCGAAATCGGTAGACGCAGTTGATTCUAAATCAACCGTAGAAATACGTGCCGGTTCGAGTCCGGCCTTCGGCACCAAATTCGAAAAGCCTGCTCAACGAGCAGGCTTTTTTGCATGGCGTTACCCAACTTAATCGCCTTGCAGCACATCCCCCTTTCGCCAGATCCCGCAAGAGGCCCGGCAGTACCGGCATAACCAAGCCTATGCCTACAGCATCCAGGGTGACGGTGCCGAGGATGACGATGAGCGCATTGTTAGATTTCATACACGGTGCCTGACTGCGTTAGCAATTTAACTGTGATAAACTACCGCATTAAAGCTTATCGATGATAAGCTGTCAAACATGAGAATTACAACTTATATCGTATGGGGCTGACTTCAGGTGCTACATTTGAAGAGATAAATTGCACTGAAATCTAGAAATATTTTATCTGATTAATAAGATGATCTTCTTGAGATCGTTTTGGTCTGCGCGTAATCTCTTGCTCTGAAAACGAAAAAACCGCCTTGCAGGGCGGTTTTTCGAAGGTTCTCTGAGCTACCAACTCTTTGAACCGAGGTAACTGGCTTGGAGGAGCGCAGTCACCAAAACTTGTCCTTTCAGTTTAGCCTTAACCGGCGCATGACTTCAAGACTAACTCCTCTAAATCAATTACCAGTGGCTGCTGCCAGTGGTGCTTTTGCATGTCTTTCCGGGTTGGACTCAAGACGATAGTTACCGGATAAGGCGCAGCGGTCGGACTGAACGGGGGGTTCGTGCATACAGTCCAGCTTGGAGCGAACTGCCTACCCGGAACTGAGTGTCAGGCGTGGAATGAGACAAACGCGGCCATAACAGCGGAATGACACCGGTAAACCGAAAGGCAGGAACAGGAGAGCGCACGAGGGAGCCGCCAGGGGGAAACGCCTGGTATCTTTATAGTCCTGTCGGGTTTCGCCACCACTGATTTGAGCGTCAGATTTCGTGATGCTTGTCAGGGGGGCGGAGCCTATGGAAAAACGGCTTTGCCGCGGCCCTCTCACTTCCCTGTTAAGTATCTTCCTGGCATCTTCCAGGAAATCTCCGCCCCGTTCGTAAGCCATTTCCGCTCGCCGCAGTCGAACGACCGAGCGTAGCGAGTCAGTGAGCGAGGAAGCGGAATATATCCTGTATCACATATTCTGCTGACGCACCGGTGCAGCCTTTTTTCTCCTGCCACATGAAGCACTTCACTGACACCCTCATCAGTGCCAACATAGTAAGCCAGTATACACTCCGCTAGCGCTGATGTCCGGCGGTGCTTTTGCCGTTACGCACCACCCCGTCAGTAGCTGAACAGGAGGGACAGCTGATAGAAACAGAAGCCACTGGAGCACCTCAAAAACACCATCATACACTAAATCAGTAAGTTGGCAGCATCACCCGACGCACTTTGCGCCGAATAAATACCTGTGACGGAAGATCACTTCGCAGAATAAATAAATCCTGGTGTCCCTGTTGATACCGGGAAGCCCTGGGCCAACTTTTGGCGAAAATGAGACGTTGATCGGCACGTAAGAGGTTCCAACTTTCACCATAATGAAATAAGATCACTACCGGGCGTATTTTTTGAGTTATCGAGATTTTCAGGAGCTAAGGAAGCTAAA

**Sequence of pJH674**

CGTATGGCAATGAAAGACGGTGAGCTGGTGATATGGGATAGTGTTCACCCTTGTTACACCGTTTTCCATGAGCAAACTGAAACGTTTTCATCGCTCTGGAGTGAATACCACGACGATTTCCGGCAGTTTCTACACATATATTCGCAAGATGTGGCGTGTTACGGTGAAAACCTGGCCTATTTCCCTAAAGGGTTTATTGAGAATATGTTTTTCGTCTCAGCCAATCCCTGGGTGAGTTTCACCAGTTTTGATTTAAACGTGGCCAATATGGACAACTTCTTCGCCCCCGTTTTCACCATGGGCAAATATTATACGCAAGGCGACAAGGTGCTGATGCCGCTGGCGATTCAGGTTCATCATGCCGTCTGTGATGGCTTCCATGTCGGCAGAATGCTTAATGAATTACAACAGTACTGCGATGAGTGGCAGGGCGGGGCGTAATTTTTTTAAGGCAGTTATTGGTGCCCTTAAACGCCTGGTGCTACGCCTGAATAAGTGATAATAAGCGGATGAATGGCAGAAATTCGAAAGCAAATTCGACCCGGGCGGCCGCAACCCAGCGTTCGATGCTTCTTTGAGCGAACGATCAAAAATAAGTGCCTTCCCATCAATTGGCCCTACCAATTCTTCGCTTATCTGACCTCTGGTTCACAATTTCCCAATTAAAACTCACATCAATGTTGCCAATACATAACATTTAGTTAACCATTCATTGTCATTATCCCTACACAACACAATTGGCAGTGCCACTTGCTATGAGCCGCTGATGAGTCCGTGAGGACGAAACGGTACCCGGTACCGTCGCCGAAGTGGCGAAATCGGTAGACGCAGTTGATTCTAAATCAACCGTAGAAATACGTGCCGGTTCGAGTCCGGCCTTCGGCACCAAATTCGAAAAGCCTGCTCAACGAGCAGGCTTTTTTGCATGGCGTTACCCAACTTAATCGCCTTGCAGCACATCCCCCTTTCGCCAGATCCCGCAAGAGGCCCGGCAGTACCGGCATAACCAAGCCTATGCCTACAGCATCCAGGGTGACGGTGCCGAGGATGACGATGAGCGCATTGTTAGATTTCATACACGGTGCCTGACTGCGTTAGCAATTTAACTGTGATAAACTACCGCATTAAAGCTTATCGATGATAAGCTGTCAAACATGAGAATTACAACTTATATCGTATGGGGCTGACTTCAGGTGCTACATTTGAAGAGATAAATTGCACTGAAATCTAGAAATATTTTATCTGATTAATAAGATGATCTTCTTGAGATCGTTTTGGTCTGCGCGTAATCTCTTGCTCTGAAAACGAAAAAACCGCCTTGCAGGGCGGTTTTTCGAAGGTTCTCTGAGCTACCAACTCTTTGAACCGAGGTAACTGGCTTGGAGGAGCGCAGTCACCAAAACTTGTCCTTTCAGTTTAGCCTTAACCGGCGCATGACTTCAAGACTAACTCCTCTAAATCAATTACCAGTGGCTGCTGCCAGTGGTGCTTTTGCATGTCTTTCCGGGTTGGACTCAAGACGATAGTTACCGGATAAGGCGCAGCGGTCGGACTGAACGGGGGGTTCGTGCATACAGTCCAGCTTGGAGCGAACTGCCTACCCGGAACTGAGTGTCAGGCGTGGAATGAGACAAACGCGGCCATAACAGCGGAATGACACCGGTAAACCGAAAGGCAGGAACAGGAGAGCGCACGAGGGAGCCGCCAGGGGGAAACGCCTGGTATCTTTATAGTCCTGTCGGGTTTCGCCACCACTGATTTGAGCGTCAGATTTCGTGATGCTTGTCAGGGGGGCGGAGCCTATGGAAAAACGGCTTTGCCGCGGCCCTCTCACTTCCCTGTTAAGTATCTTCCTGGCATCTTCCAGGAAATCTCCGCCCCGTTCGTAAGCCATTTCCGCTCGCCGCAGTCGAACGACCGAGCGTAGCGAGTCAGTGAGCGAGGAAGCGGAATATATCCTGTATCACATATTCTGCTGACGCACCGGTGCAGCCTTTTTTCTCCTGCCACATGAAGCACTTCACTGACACCCTCATCAGTGCCAACATAGTAAGCCAGTATACACTCCGCTAGCGCTGATGTCCGGCGGTGCTTTTGCCGTTACGCACCACCCCGTCAGTAGCTGAACAGGAGGGACAGCTGATAGAAACAGAAGCCACTGGAGCACCTCAAAAACACCATCATACACTAAATCAGTAAGTTGGCAGCATCACCCGACGCACTTTGCGCCGAATAAATACCTGTGACGGAAGATCACTTCGCAGAATAAATAAATCCTGGTGTCCCTGTTGATACCGGGAAGCCCTGGGCCAACTTTTGGCGAAAATGAGACGTTGATCGGCACGTAAGAGGTTCCAACTTTCACCATAATGAAATAAGATCACTACCGGGCGTATTTTTTGAGTTATCGAGATTTTCAGGAGCTAAGGAAGCTAAAATGGAGAAAAAAATCACTGGATATACCACCGTTGATATATCCCAATGGCATCGTAAAGAACATTTTGAGGCATTTCAGTCAGTTGCTCAATGTACCTATAACCAGACCGTTCAGCTGGATATTACGGCCTTTTTAAAGACCGTAAAGAAAAATAAGCACAAGTTTTATCCGGCCTTTATTCACATTCTTGCCCGCCTGATGAATGCTCATCCGGAACTC

**Sequence of pJH625**

TCCGCTCATGAGACAATAACCCTGATAAATGCTTCAATAATATTGAAAAAGGAAGAGTATGAGTATTCAACATTTCCGTGTCGCCCTTATTCCCTTTTTTGCGGCATTTTGCCTTCCTGTTTTTGCTCACCCAGAAACGCTGGTGAAAGTAAAAGATGCTGAAGATCAGTTGGGTGCACGAGTGGGTTACATCGAACTGGATCTCAACAGCGGTAAGATCCTTGAGAGTTTTCGCCCCGAAGAACGTTTTCCAATGATGAGCACTTTTAAAGTTCTGCTATGTGGCGCGGTATTATCCCGTATTGACGCCGGGCAAGAGCAACTCGGTCGCCGCATACACTATTCTCAGAATGACTTGGTTGAGTACTCACCAGTCACAGAAAAGCATCTTACGGATGGCATGACAGTAAGAGAATTATGCAGTGCTGCCATAACCATGAGTGATAACACTGCGGCCAACTTACTTCTGACAACGATCGGAGGACCGAAGGAGCTAACCGCTTTTTTGCACAACATGGGGGATCATGTAACTCGCCTTGATCGTTGGGAACCGGAGCTGAATGAAGCCATACCAAACGACGAGCGTGACACCACGATGCCTGTAGCAATGGCAACAACGTTGCGCAAACTATTAACTGGCGAACTACTTACTCTAGCTTCCCGGCAACAATTGATAGACTGGATGGAGGCGGATAAAGTTGCAGGACCACTTCTGCGCTCGGCCCTTCCGGCTGGCTGGTTTATTGCTGATAAATCTGGAGCCGGTGAGCGTGGCTCTCGCGGTATCATTGCAGCACTGGGGCCAGATGGTAAGCCCTCCCGTATCGTAGTTATCTACACGACGGGGAGTCAGGCAACTATGGATGAACGAAATAGACAGATCGCTGAGATAGGTGCCTCACTGATTAAGCATTGGTAACTGTCAGACCAAGTTTACTCATATATACTTTAGATTGATTTAAAACTTCATTTTTAATTTAAAAGGATCTAGGTGAAGATCCTTTTTGATAATCTCATGACCAAAATCCCTTAACGTGGGTTTTCGTTCCACTGAGCGTCAGACCCCGTAGAAAAGATCAAAGGATCATCTTGAGATCCTTTTTTTCTGCGCGTAATCTGCTGCTTGCAAACAAAAAAACCACCGCTACCAGCGGTGGTTTGTTTGCCGGATCAAGAGCTACCAACTCTTTTTCCGAAGGTAACTGGCTTCAGCAGAGCGCAGATACCAAATACTGTTCTTCTAGTGTAGCCGTAGTTAGGCCACCACTTCAAGAACTCTGTAGCACCGCCTACATACCTCGCTCTGCTAATCCTGTTACCAGTGGCTGCTGCCAGTGGCGATAAGTCGTGTCTTACCGGGTTGGACTCAAGACGATAGTTACCGGATAAGGCGCAGCGGTCGGGCTGAACGGGGGGTTCGTGCACACAGCCCAGCTTGGAGCGAACGACCTACACCGAACTGAGATACCTACAGCGTGAGCTATGAGAAAGCGCCACGCTTCCCGAAGGGAGAAAGGCGGACAGGTATCCGGTAAGCGGCAGGGTCGGAACAGGAGAGCGCACGAGGGAGCTTCCAGGGGGAAACGCCTGGTATCTTTATAGTCCTGTCGGGTTTCGCCACCTCTGACTTGAGCGTCGATTTTTGTGATGCTCGTCAGGGGGGCGGAGCCTATGGAAAAACGCCAGCAACGCGGCCTTTTTACGGTTCCTGGCCTTTTGCTGGCCTTTTGCTCAGGCTGTTTTGGCGGATGAGAGAAGATTTTCAGCCTGATACAGATTAAATCAGAACGCAGAAGCGGTCTGATAAAACAGAATTTGCCTGGCGGCAGTAGCGCGGTGGTCCCACCTGACCCCATGCCGAACTCAGAAGTGAAACGCCGTAGCGCCGATGGTAGTGTGGGGTCTCCCCATGCGAGAGTAGGGAACTGCCAGGCATCAAATAAAACGAAAGGCTCAGTCGAAAGACTGGGCCTTTCGTTTTATCTGTTGTTTGTCGGTGAACGCTCTCCTGAGTAGGACAAATCCGCCGGGAGCGGATTTGAACGTTGCGAAGCAACGGCCCGGAGGGTGGCGGGCAGGACGCCCGCCATAAACTGCCAGGCATCAAATTAAGCAGAAGGCCATCCTGACGGATGGCCTTTTTGCGTTTCTACAAACTCTGCTAGCAAGTAAGGCCGACCAACAACCTAAGGGCAATTCTCTGATGAGGATTGCCCTTTTCTTTACCAGACATCTCCCCCCACAAGAATTGGCCCTACCAATTCTTCGCTTATCTGACCTCTGGTTCACAATTTCCCAATTAAAACTCACATCAATGTTGCCAATACATAACATTTAGTTAACCATTCATTGTCATTATCCCTACACAACACAATTGGCAGTGCCACTTTTACACAACGTGTGACAAGGAGATGAGCAACAGACTCATTACACGATGTGCGTGGACTCCCATTTAACTTTAAGAAGGAGATATACATATGCGTAAAGGTGAAGAACTGTTCACCGGTGTTGTTCCGATCCTGGTTGAACTGGACGGTGACGTTAACGGTCACAAATTCTCTGTTCGTGGTGAAGGTGAAGGTGACGCTACCAACGGTAAACTGACCCTGAAATTCATCTGCACCACCGGTAAACTGCCGGTTCCGTGGCCGACCCTGGTTACCACCCTGACCTACGGTGTTCAGTGCTTCGCTCGTTACCCGGACCACATGAAACAGCACGACTTCTTCAAATCTGCTATGCCGGAAGGTTACGTTCAGGAACGTACCATCTCTTTCAAAGACGACGGTACCTACAAAACCCGTGCTGAAGTTAAATTCGAAGGTGACACCCTGGTTAACCGTATCGAACTGAAAGGTATCGACTTCAAAGAAGACGGTAACATCCTGGGTCACAAACTGGAATACAACTTCAACTCTCACAACGTTTACATCACCGCTGACAAACAGAAAAACGGTATCAAAGCTAACTTCAAAATCCGTCACAACGTTGAAGACGGTTCTGTTCAGCTGGCTGACCACTACCAGCAGAACACCCCGATCGGTGACGGTCCGGTTCTGCTGCCGGACAACCACTACCTGTCTACCCAGTCTGTTCTGTCTAAAGACCCGAACGAAAAACGTGACCACATGGTTCTGCTGGAATTCGTTACCGCTGCTGGTATCACCCACGGTATGGACGAACTGTACAAAGATGCATGCCAGTTCTAACATAACCCTAATGAGTGAGCTAACTTACATTAATTGCGTTGCGCCTTAATTAACGGCACTCCTCAGCAAATATAATGACCCTCTTGATAACCCAAGAGGGCATTTTTTAATGCCCATGGCGTTTACCACAGCTAACACCACGTCGTCCCTATCTGCTGCCCTAGGTCTATGAGTGGTTGCTGGATAACTTTACGGGCATGCATAAGGCTCGTAGGCTATATTCAGGGAGACCACAACGGTTTCCCTCTACAAATAATTTTGTTTAACTTTGAAATAAGGAGGTAATACAAATGATTGTTTTACCCAGACGCCTGTCAGACGAGGTTGCCGATCGTGTGCGGGCGCTGATTGATGAAAAAAACCTGGAAGCGGGCATGAAGTTGCCCGCTGAGCGCCAACTGGCGATGCAACTCGGCGTATCACGTAATTCACTGCGCGAGGCGCTGGCAAAACTGGTGAGTGAAGGCGTGCTGCTCAGTCGACGCGGCGGCGGGACGTTTATTCGCTGGCGTCATGACACATGGTCGGAGCAAAACATCGTCCAGCCGCTAAAAACACTGATGGCCGATGATCCGGATTACAGTTTCGATATTCTGGAAGCCCGCTACGCCATTGAAGCCAGCACCGCATGGCATGCGGCAATGCGCGCCACACCTGGCGACAAAGAAAAGATTCAGCTTTGCTTTGAAGCAACGCTAAGTGAAGACCCGGATATCGCCTCACAAGCGGACGTTCGTTTTCATCTGGCGATTGCCGAAGCCTCACATAACATCGTGCTGCTGCAAACCATGCGCGGTTTCTTCGATGTCCTGCAATCCTCAGTGAAGCATAGCCGTCAGCGGATGTATCTGGTGCCACCGGTTTTTTCACAACTGACCGAACAACATCAGGCTGTCATTGACGCCATTTTTGCCGGTGATGCTGACGGGGCGCGTAAAGCAATGATGGCGCACCTTAGTTTTGTTCACACCACCATGAAACGATTCGATGAAGATCAGGCTCGCCACGCACGGATTACCCGCCTGCCCGGTGAGCATAATGAGCATTCGAGGGAGAAAAACGCATAATAAGCGACTAAAAAATTGAATGTAGGAAACCAACATGCCAGTTCGAGCAATAACTAGCATAACCCCTTGGGGCCTCTAAACGGGTCTTGAGGGGTTTTTTGCTGAAACCTCAGGCATTTGAGAAGCACACGGTCACACT

**Sequence of MP6.6TAG**

CACTCGGTCGCTACGCTCCGGGCGTGAGACTGCGGCGGGCGCTGCGGACACATACAAAGTTACCCACAGATTCCGTGGATAAGCAGGGGACTAACATGTGAGGCAAAACAGCAGGGCCGCGCCGGTGGCGTTTTTCCATAGGCTCCGCCCTCCTGCCAGAGTTCACATAAACAGACGCTTTTCCGGTGCATCTGTGGGAGCCGTGAGGCTCAACCATGAATCTGACAGTACGGGCGAAACCCGACAGGACTTAAAGATCCCCACCGTTTCCGGCGGGTCGCTCCCTCTTGCGCTCTCCTGTTCCGACCCTGCCGTTTACCGGATACCTGTTCCGCCTTTCTCCCTTACGGGAAGTGTGGCGCTTTCTCATAGCTCACACACTGGTATCTCGGCTCGGTGTAGGTCGTTCGCTCCAAGCTGGGCTGTAAGCAAGAACTCCCCGTTCAGCCCGACTGCTGCGCCTTATCCGGTAACTGTTCACTTGAGTCCAACCCGGAAAAGCACGGTAAAACGCCACTGGCAGCAGCCATTGGTAACTGGGAGTTCGCAGAGGATTTGTTTAGCTAAACACGCGGTTGCTCTTGAAGTGTGCGCCAAAGTCCGGCTACACTGGAAGGACAGATTTGGTTGCTGTGCTCTGCGAAAGCCAGTTACCACGGTTAAGCAGTTCCCCAACTGACTTAACCTTCGATCAAACCACCTCCCCAGGTGGTTTTTTCGTTTACAGGGCAAAAGATTACGCGCAGAAAAAAAGGATCTCAAGAAGATCCTTTGATCTTTTCTACTGAACCGCTCTAGATTTCAGTGCAATTTATCTCTTCAAATGTAGCACCTGAAGTCAGCCCAGGAGGAAGAGGACATCCGGTCAAATAAAACGAAAGGCTCAGTCGAAAGACTGGGCCTTTCGTTTTAGACTTAGGGACCCTTTATGACAACTTGACGGCTACATCATTCACTTTTTCTTCACAACCGGCACGGAACTCGCTCGGGCTGGCCCCGGTGCATTTTTTAAATACCCGCGAGAAATAGAGTTGATCGTCAAAACCAACATTGCGACCGACGGTGGCGATAGGCATCCGGGTGGTGCTCAAAAGCAGCTTCGCCTGGCTGATACGTTGGTCCTCGCGCCAGCTTAAGACGCTAATCCCTAACTGCTGGCGGAAAAGATGTGACAGACGCGACGGCGACAAGCAAACATGCTGTGCGACGCTGGCGATATCAAAATTGCTGTCTGCCAGGTGATCGCTGATGTACTGACAAGCCTCGCGTACCCGATTATCCATCGGTGGATGGAGCGACTCGTTAATCGCTTCCATGCGCCGCAGTAACAATTGCTCAAGCAGATTTATCGCCAGCAGCTCCGAATAGCGCCCTTCCCCTTGCCCGGCGTTAATGATTTGCCCAAACAGGTCGCTGAAATGCGGCTGGTGCGCTTCATCCGGGCGAAAGAACCCCGTATTGGCAAATATTGACGGCCAGTTAAGCCATTCATGCCAGTAGGCGCGCGGACGAAAGTAAACCCACTGGTGATACCATTCGCGAGCCTCCGGATGACGACCGTAGTGATGAATCTCTCCTGGCGGGAACAGCAAAATATCACCCGGTCGGCAAACAAATTCTCGTCCCTGATTTTTCACCACCCCCTGACCGCGAATGGTGAGATTGAGAATATAACCTTTCATTCCCAGCGGTCGGTCGATAAAAAAATCGAGATAACCGTTGGCCTCAATCGGCGTTAAACCCGCCACCAGATGGGCATTAAACGAGTATCCCGGCAGCAGGGGATCATTTTGCGCTTCAGCCATACTTTTCATACTCCCACCATTCAGAGAAGAAACCAATTGTCCATATTGCATCAGACATTGCCGTCACTGCGTCTTTTACTGGCTCTTCTCGCTAACCCAACCGGTAACCCCGCTTATTAAAAGCATTCTGTAACAAAGCGGGACCAAAGCCATGACAAAAACGCGTAACAAAAGTGTCTATAATCACGGCAGAAAAGTCCACATTGATTATTTGCACGGCGTCACACTTTGCTATGCCATAGCATTTTTATCCATAAGATTAGCGGATCCTACCTGACGCTTTTTATCGCAACTCTCTACTGTTTCTCCATACCCGTTTTTTTGGACGCGTACAACTCAAGTCTGACATAAATGACCGCTATGTAGAGCACTGCAATTACACGCCAGATCGTTCTCGCTACCGCAACCACCGGTATGAACCAGATTGGTGCGCACTATGAAGGCCACAAGATCATTGAGATTGGTGCCGTTGAAGTGGTGAACCGTCGCCTGACGGGCAATAACTTCCATGTTTATCTCAAACCCGATCGGCTGGTGGATCCGGAAGCCTTTGGCGTACATGGTATTGCCGATGAATTTTTGCTCGATAAGCCCACGTTTGCCGAAGTAGCCGATGAGTTCATGGACTATATTCGCGGCGCGGAGTTGGTGATCCATAACGCAGCGTTCGATATCGGCTTTATGGACTACGAGTTTTCGTTGCTTAAGCGCGATATTCCGAAGACCAATACTTTCTGTAAGGTCACCGATAGCCTTGCGGTGGCGAGGAAAATGTTTCCCGGTAAGCGCAACAGCCTCGATGCGTTATGTGCTCGCTACGAAATAGATAACAGTAAACGAACGCTGCACGGGGCATTACTCGATGCCCAGATCCTTGCGGAAGTTTATCTGGCGATGACCGGTGGTCAAACGTCGATGGCTTTTGCGATGGAAGGAGAGACACAACAGCAACAAGGTGAAGCAACAATTCAGCGCATTGTACGTCAGGCAAGTAAGTTACGCGTTGTTTTTGCGACAGATGAAGAGATTGCAGCTCATGAAGCCCGTCTCGATCTGGTGCAGAAGAAAGGCGGAAGTTGCCTCTGGCGAGCATAATTTAATATCAGTAAACCGGACATAACCCATGTAGAAGAAAAATCGCGCTTTTTTGAAGTGGGCAGGGGGCAAGTATCCCCTGCTTGATGATATTAAACGGCATTTGCCCAAGGGCGAATGTCTGGTTGAGCCTTTTGTAGGTGCCGGGTCGGTGTTTCTCAACACCGACTTTTCTCGTTATATCCTTGCCGATATCAATAGCGACCTGATCAGTCTCTATAACATTGTGAAGATGCGTACTGATGAGTACGTACAGGCCGCACGCGAGCTGTTTGTTCCCGAAACAAATTGCGCCGAGGTTTACTATCAGTTCCGCGAAGAGTTCAACAAAAGCCAGGATCCGTTCCGTCGGGCGGTACTGTTTTTATATTTGAACCGCTACGGTTACAACGGCCTGTGTCGTTACAATCTGCGCGGTGAGTTTAACGTGCCGTTCGGCCGCTACAAAAAACCCTATTTCCCGGAAGCAGAGTTGTATCACTTCGCTGAAAAAGCGCAGAATGCCTTTTTCTATTGTGAGTCTTACGCCGATAGCATGGCGCGCGCAGATGATGCATCCGTCGTCTATTGCGATCCGCCTTATGCACCGCTGTCTGCGACCGCCAACTTTACGGCGTATCACACAAACAGTTTTACGCTTGAACAACAAGCGCATCTGGCGGAGATCGCCGAAGGTCTGGTTGAGCGCCATATTCCAGTGCTGATCTCCAATCACGATACGATGTTAACGCGTGAGTGGTATCAGCGCGCAAAATTGCATGTCGTCAAAGTTCGACGCAGTATAAGCAGCAACGGCGGCACACGTAAAAAGGTGGACGAACTGCTGGCTTTGTACAAACCAGGAGTCGTTTCACCCGCGAAAAAATAATTCAGCTAAGACACTGCACTGGATTAAGATGTAGAAAACGATTGAAGTTGATGATGAACTCTACAGCTATATTGCCAGCCACACTAAGCATATCGGCGAGAGCGCATCCGACATTTTACGGCGTATGTTGAAATTTTCCGCCGCATCACAGCCTGCTGCTCCGGTGACGAAAGAGGTTCGCGTTGCGTCACCTGCTATCGTCGAAGCGAAGCCGGTCAAAACGATTAAAGACAAGGTTCGCGCAATGCGTGAACTTCTGCTTTCGGATGAATACGCAGAGCAAAAGCGAGCGGTCAATCGCTTTATGCTGCTGTTGTCTACACTATATTCTCTTGACGCCCAGGCGTTTGCCGAAGCAACGGAATCGTTGCACGGTCGTACACGCGTTTACTTTGCGGCAGATGAACAAACGCTGCTGAAAAATGGTAATCAGACCAAGCCGAAACATGTGCCAGGCACGCCGTATTGGGTGATCACCAACACCAACACCGGCCGTAAATGCAGCATGATCGAACACATCATGCAGTCGATGCAATTCCCGGCGGAATTGATTGAGAAGGTTTGCGGAACTATCTAACGGCTGAAATTAATGAGGTCATACCCAAATGTAGGATAGTTCGTTTACGCCCATTGAACAAATGCTAAAATTTCGCGCCAGCCGCCACGAAGATTTTCCTTATCAGGAGATCCTTCTGACTCGTCTTTGCATGCACATGCAAAGCAAGCTGCTGGAGAACCGCAATAAAATGCTGAAGGCTCAGGGAATTAACGAGACGTTGTTTATGGCGTTGATTACGCTGGAGTCTCAGGAAAACCACAGTATTCAGCCTTCTGAATTAAGTTGTGCTCTTGGATCATCCCGTACCAACGCGACGCGTATTGCCGATGAACTGGAAAAACGCGGTTGGATCGAACGTCGTGAAAGCGATAACGATCGCCGCTGCCTGCATCTGCAATTAACGGAAAAAGGTCACGAGTTTTTGCGCGAGGTTTTACCACCGCAGCATAACTGCCTGCATCAACTCTGGTCCGCGCTCAGCACAACAGAAAAAGATCAGCTCGAGCAAATCACCCGCAAATTGCTCTCCCGTCTCGACCAGATGGAACAAGACGGTGTGGTTCTCGAAGCGATGAGCTAATAATACAAAAATTAGGAGGAATTTCAACATGTAGACAAATTTATCTGACATCATTGAAAAAGAAACAGGAAAACAACTAGTGATTCAAGAATCAATTCTAATGTTACCAGAAGAAGTAGAGGAAGTAATTGGGAATAAACCAGAAAGTGATATTTTAGTTCATACTGCTTATGATGAAAGTACAGATGAAAATGTAATGCTATTAACTTCAGATGCTCCAGAATATAAACCTTGGGCTTTAGTAATTCAAGACAGTAATGGAGAAAATAAAATTAAAATGTTATAAGTCGAGATTAAGTAAACCGGAATCTGAAGATGTAGACCGACGCGGAATACGTTCGTATCCACGAAAAACTGGACATCTACACCTTCAAAAAACAGTTCTTCAACAACAAAAAATCTGTTTCTCACCGTTGCTACGTTCTGTTCGAACTGAAACGTCGTGGTGAACGTCGTGCGTGCTTCTGGGGTTACGCGGTTAACAAACCGCAGTCTGGTACCGAACGTGGTATCCACGCGGAAATCTTCTCTATCCGTAAAGTTGAAGAATACCTGCGTGACAACCCGGGTCAGTTCACCATCAACTGGTACTCTTCTTGGTCTCCGTGCGCGGACTGCGCGGAAAAAATCCTGGAATGGTACAACCAGGAACTGCGTGGTAACGGTCACACCCTGAAAATCTGGGCGTGCAAACTGTACTACGAAAAAAACGCGCGTAACCAGATCGGTCTGTGGAACCTGCGTGACAACGGTGTTGGTCTGAACGTTATGGTTTCTGAACACTACCAGTGCTGCCGTAAAATCTTCATCCAGTCTTCTCACAACCAGCTGAACGAAAACCGTTGGCTGGAAAAAACCCTGAAACGTGCGGAAAAACGTCGTTCTGAACTGTCTATCATGATCCAGGTTAAAATCCTGCACACCACCAAATCTCCGGCGGTTTAAACTTAATTAACGGCACTCCTCAGCCAAGTCAAAAGCCTCCGGTCGGAGGCTTTTGACTACATGCCCATGGCGTTTACGCCCCGCCCTGCCACTCATCGCAGTACTGTTGTAATTCATTAAGCATTCTGCCGACATGGAAGCCATCACAAACGGCATGATGAACCTGAATCGCCAGCGGCATCAGCACCTTGTCGCCTTGCGTATAATATTTGCCCATAGTGAAAACGGGGGCGAAGAAGTTGTCCATATTGGCCACGTTTAAATCAAAACTGGTGAAACTCACCCAGGGATTGGCTGAGACGAAAAACATATTCTCAATAAACCCTTTAGGGAAATAGGCCAGGTTTTCACCGTAACACGCCACATCTTGCGAATATATGTGTAGAAACTGCCGGAAATCGTCGTGGTATTCACTCCAGAGCGATGAAAACGTTTCAGTTTGCTCATGGAAAACGGTGTAACAAGGGTGAACACTATCCCATATCACCAGCTCACCGTCTTTCATTGCCATACGGAACTCCGGATGAGCATTCATCAGGCGGGCAAGAATGTGAATAAAGGCCGGATAAAACTTGTGCTTATTTTTCTTTACGGTCTTTAAAAAGGCCGTAATATCCAGCTGAACGGTCTGGTTATAGGTACATTGAGTAACTGACTGAAATGCCTCAAAATGTTCTTTACGATGCCATTGGGATATATCAACGGTGGTATATCCAGTGATTTTTTTCTCCATTTTAGCTTCCTTAGCTCCTGAAAATCTCGATAACTCAAAAAATACGCCCGGTAGTGATCTTATTTCATTATGGTGAAAGTTGGAACCTCTTACGTGCCAAGCCAAATAGGCCGT

**II. Supplementary Figures**

**Fig. S1. Architecture-independent induction of functional supP**. (A) Transcriptional repression by LldR transcription factor requires the LldRO2 operator site to be positioned after the transcription start site, which leaves an unwanted 5’ appendage on the transcript. (B) Installation of a sequence encoding a self-cleaving hammerhead ribozyme (HHRz) between the LldRO2 operator site and *supP* results in cleavage at the 3’ end of the HHRz RNA, thereby correctly processing the 5’ end of the downstream tRNA and yielding mature functional tRNA^Leu^. As the function of HHRz is unaffected by the addition of nucleotides to its 5’ end, this HHRz-*supP* design makes our tool compatible with any arbitrary promoter or inducible genetic system, regardless of variations in transcription start sites (C) Predicted secondary structure of the full-length transcript shows physically separate intact structures of the scar, HHRz, and tRNA.

**Fig. S2. LldR regulation of GFP through conditional translation alone**. Induction of *supP* was tested across multiple concentrations of lactate while GFP variants with varying numbers of amber codons were expressed from a constitutive promoter in *E. coli* C321.ΔA.exp. (A) Fluorescence/A600 response curves for each GFP variant are shown as a function of inducer concentration. (B) Fold-change induction is shown for each GFP variant, calculated by dividing fluorescence/A600 in the presence of 100 mM lactate by the observed fluorescence/A600 in the absence of lactate. (C) Readthrough (amber suppression) efficiency is shown for each GFP variant at each lactate concentration, calculated by dividing fluorescence/A600 signal observed for each GFP variant by the signal observed for GFP containing 0 stop codons. Read-through efficiency increases with inducer concentration, mediated by increased expression of *supP*. (D) Leaky gene expression, defined as fluorescence/A600 signal observed in the absence of lactate inducer, is shown as a function of the number of amber codons present in regulated GFP variants. Data shows the average across three biological replicates, and error bars show the standard deviation.

**Fig. S3. Growth curves and fluorescence response for the best variants in Turbo, with and without expression of *supP***. The growth *rate* of Turbo encoding the amber leak dampener is slower than that of Turbo alone, but all samples reaches the same *final* cell density, i.e. Turbo alone reaches stationary phase before the other samples, but all samples reach the same A_600_ at stationary phase. All fluorescent measurements were taken after resuspending the cells at the final timepoint (18 h post-induction); since the A_600_ values at the final timepoint are comparable (see, final A_600_ values of resuspended samples in Tables S3 and S4), fluorescence/A_600_ is a fair metric to use for characterizing functionality of our leak dampener tool.

**Fig. S4. Leak dampener tool applied to AraC induction using amber codons in *E. coli* S1030 containing RF1**. Induction of *supP* was tested across multiple concentrations of arabinose while GFP variants with varying numbers of amber codons were expressed from an arabinose-inducible promoter in *E. coli* S1030. (A) Fluorescence/A600 response curves for each GFP variant are shown as a function of inducer concentration, with high signal retention (88–94%) at 100 mM lactate. (B) Fold-change induction is shown for each GFP variant, calculated by dividing fluorescence/A600 in the presence of 1 mM arabinose by the observed fluorescence/A600 in the absence of arabinose. A 1.5–1.7-fold improvement in fold-change induction is observed for all cases. (C) Readthrough (amber suppression) efficiency is shown for each GFP variant at each arabinose concentration, calculated by dividing fluorescence/A600 signal observed for each GFP variant by the signal observed for GFP containing 0 stop codons. Read-through efficiency increases with inducer concentration, mediated by increased expression of *supP*. (D) Leaky gene expression, defined as fluorescence/A600 signal observed in the absence of arabinose inducer, is shown as a function of the number of amber codons present in regulated GFP variants and is reduced by 1.6–1.9-fold. Data shows the average across two biological replicates, and error bars show the standard deviation.

**III. Supplementary Tables**

**Table S1. List of constructs used in this study**. MP6 (Addgene #69669) was a generous gift from Prof. David Liu. New constructs are available in the public repository Addgene.

| **Plasmid** | **Description** | **Insert** | **Resistance** | **Origin** | **Addgene** |
| --- | --- | --- | --- | --- | --- |
| pJH474 | pTech_Para-supP | supP | Kan | p15A | 163718 |
| MP6 | AraC_Para-dnaQ926-dam-seqA-emrR-ugi-CDA1 | araC dnaQ926 dam seqA emrR ugi CDA1 (Badran and Liu, 2015) | Chl | CloDF13 | Gift |
| MP6.6TAG | AraC_Para-dnaQ926-dam-seqA-emrR-ugi-CDA1.6TAG | araC dnaQ926.1TAG dam.1TAG seqA.1TAG emrR.1TAG ugi.1TAG CDA1.1TAG | Chl | CloDF13 | 163719 |
| pJH25 | pBad_AraC_Para-sfGFP.0TAG | araC gfp | Amp | pBR322 | 163720 |
| pJH26 | pBad_AraC_Para-sfGFP.1TAG | araC gfp.1TAG | Amp | pBR322 | 163721 |
| pJH27 | pBad_AraC_Para-sfGFP.2TAG | araC gfp.2TAG | Amp | pBR322 | 163722 |
| pJH28 | pBad_AraC_Para-sfGFP.3TAG | araC gfp.3TAG | Amp | pBR322 | 163723 |
| pJH625 | pJH_LldR_PlldR-sfGFP.0TAG | lldr gfp | Amp | pBR322 | 163724 |
| pJH626 | pJH_LldR_PlldR-sfGFP.1TAG | lldr gfp.1TAG | Amp | pBR322 | 163725 |
| pJH627 | pJH_LldR_PlldR-sfGFP.2TAG | lldr gfp.2TAG | Amp | pBR322 | 163726 |
| pJH628 | pJH_LldR_PlldR-sfGFP.3TAG | lldr gfp.3TAG | Amp | pBR322 | 163727 |
| pJH674 | pTech_PlldR-HHRz-supP | hhrz-supP | Chl | p15A | 163728 |
| pJH721 | pJH_LldR_PlldR-sfGFP.20TAG | lldr gfp.20TAG | Amp | pBR322 | 163729 |
| pJH726 | pJH_LldR_PlldR-sfGFP.15TAG | lldr gfp.15TAG | Amp | pBR322 | 163730 |
| pJH727 | pJH_LldR_PlldR-sfGFP.10TAG | lldr gfp.10TAG | Amp | pBR322 | 163731 |
| pJH773 | pTech_PlldR-HHRz-supP(opal) | hhrz-supP | Chl | p15A | 163732 |
| pJH774 | pJH_LldR_PlldR-sfGFP.1opal | lldr gfp.1TGA | Amp | pBR322 | 163733 |
| pJH775 | pJH_LldR_PlldR-sfGFP.2opal | lldr gfp.2TGA | Amp | pBR322 | 163734 |
| pJH776 | pJH_LldR_PlldR-sfGFP.3opal | lldr gfp.3TGA | Amp | pBR322 | 163735 |
| pJH805 | pJH_LldR_Pcon-sfGFP.0TAG | lldr gfp | Amp | pBR322 | 163736 |
| pJH806 | pJH_LldR_Pcon-sfGFP.1TAG | lldr gfp.1TAG | Amp | pBR322 | 163737 |
| pJH807 | pJH_LldR_Pcon-sfGFP.2TAG | lldr gfp.2TAG | Amp | pBR322 | 163738 |
| pJH809 | pJH_LldR_Pcon-sfGFP.10TAG | lldr gfp.10TAG | Amp | pBR322 | 163739 |

**Table S2. Fluorescence readthrough assay data for the leak dampener tool applied to LldR induction using amber codons in *E. coli* C321.ΔA.exp**. Data shown in Fig. 2A is tabulated here. For each GFP sample containing the indicated number of TAG codons, fluorescence/A600 values are shown for the average of three biological replicates alongside the corresponding standard deviation values.

| A600 (a.u.) | Average | StDev | Average | StDev | Average | StDev | Average | StDev | Average | StDev | Average | StDev | Average | StDev | Average | StDev |
| --- | --- | --- | --- | --- | --- | --- | --- | --- | --- | --- | --- | --- | --- | --- | --- | --- |
| [Lactate]/ mM | 0TAG | | 1TAG | | 2TAG | | 3TAG | | 10TAG | | 15TAG | | 20TAG | | C321.ΔA.exp | |
| 100 | 1.3615 | 0.0197 | 1.3382 | 0.0256 | 1.3155 | 0.0196 | 1.2919 | 0.0217 | 1.3364 | 0.0214 | 1.2933 | 0.0308 | 1.3422 | 0.0809 | 1.5229 | 0.0081 |
| 10 | 1.4860 | 0.0089 | 1.4815 | 0.0127 | 1.4843 | 0.0079 | 1.4889 | 0.0079 | 1.4876 | 0.0177 | 1.4730 | 0.0205 | 1.4492 | 0.0532 | 1.5429 | 0.0125 |
| 1 | 1.4726 | 0.0200 | 1.4731 | 0.0194 | 1.4751 | 0.0188 | 1.4746 | 0.0161 | 1.4883 | 0.0247 | 1.4890 | 0.0191 | 1.4449 | 0.0575 | 1.5481 | 0.0145 |
| 0 | 1.4728 | 0.0305 | 1.4747 | 0.0345 | 1.4679 | 0.0204 | 1.4743 | 0.0282 | 1.4846 | 0.0380 | 1.4937 | 0.0241 | 1.4449 | 0.0514 | 1.5490 | 0.0245 |
|  |  |  |  |  |  |  |  |  |  |  |  |  |  |  |  |  |
| Fluorescence/ A600 (a.u.) | Average | StDev | Average | StDev | Average | StDev | Average | StDev | Average | StDev | Average | StDev | Average | StDev | Average | StDev |
| [Lactate]/ mM | 0TAG | | 1TAG | | 2TAG | | 3TAG | | 10TAG | | 15TAG | | 20TAG | | C321.ΔA.exp | |
| 100 | 25595.6 | 2107.6 | 27900.9 | 2190.9 | 28258.8 | 1785.3 | 32127.3 | 1937.2 | 25723.3 | 2654.9 | 27900.4 | 1107.6 | 21747.5 | 5195.2 | 46.6 | 2.3 |
| 10 | 23910.7 | 558.5 | 25607.0 | 267.0 | 25903.7 | 117.0 | 28291.1 | 515.7 | 20443.9 | 939.6 | 19487.5 | 451.9 | 17211.3 | 1651.7 | 39.7 | 1.1 |
| 1 | 5315.3 | 173.8 | 4852.6 | 175.1 | 5185.4 | 130.6 | 5668.2 | 358.7 | 2841.3 | 562.2 | 2098.9 | 115.2 | 2256.0 | 201.3 | 37.2 | 1.7 |
| 0 (Leak) | 1286.0 | 79.8 | 1276.0 | 63.2 | 1231.0 | 61.6 | 1320.7 | 86.0 | 523.8 | 51.9 | 407.6 | 34.1 | 441.8 | 42.5 | 35.5 | 1.9 |
|  |  |  |  |  |  |  |  |  |  |  |  |  |  |  |  |  |
| Fold-change induction | Average | StDev | Average | StDev | Average | StDev | Average | StDev | Average | StDev | Average | StDev | Average | StDev |  |  |
|  | 0TAG | | 1TAG | | 2TAG | | 3TAG | | 10TAG | | 15TAG | | 20TAG | |  |  |
|  | 19.89 | 1.12 | 21.84 | 0.90 | 22.95 | 0.63 | 24.34 | 10.64 | 49.09 | 5.32 | 68.72 | 5.41 | 48.79 | 8.21 |  |  |
|  |  |  |  |  |  |  |  |  |  |  |  |  |  |  |  |  |
| Readthrough efficiency (%) | Average | StDev | Average | StDev | Average | StDev | Average | StDev | Average | StDev | Average | StDev |  |  |  |  |
| [Lactate]/ mM | 1TAG | | 2TAG | | 3TAG | | 10TAG | | 15TAG | | 20TAG | |  |  |  |  |
| 100 | 109.01 | 8.56 | 110.40 | 6.97 | 125.52 | 7.57 | 100.50 | 10.37 | 109.00 | 4.33 | 84.97 | 20.30 |  |  |  |  |
| 10 | 107.09 | 1.12 | 108.34 | 0.49 | 118.32 | 2.16 | 85.50 | 3.93 | 81.50 | 1.89 | 71.98 | 6.91 |  |  |  |  |
| 1 | 91.29 | 3.29 | 97.56 | 2.46 | 106.64 | 6.75 | 53.46 | 10.58 | 39.49 | 2.17 | 42.44 | 3.79 |  |  |  |  |
| 0 | 99.22 | 4.92 | 95.73 | 4.79 | 102.70 | 6.68 | 40.73 | 4.04 | 31.69 | 2.65 | 34.35 | 3.30 |  |  |  |  |

**Table S3. Fluorescence readthrough assay data for the leak dampener tool applied to LldR induction using amber codons in *E. coli* NEB Turbo**. Data shown in Fig. 3A is tabulated here. For each GFP sample containing the indicated number of TAG codons, fluorescence/A600 values are shown for the average of three biological replicates alongside the corresponding standard deviation values.

| A600 | Average | StDev | Average | StDev | Average | StDev | Average | StDev | Average | StDev | Average | StDev | Average | StDev |
| --- | --- | --- | --- | --- | --- | --- | --- | --- | --- | --- | --- | --- | --- | --- |
| [Lactate]/ mM | 0TAG | | 1TAG | | 2TAG | | 3TAG | | 10TAG | | 15TAG | | Turbo | |
| 100 | 1.63680 | 0.04041 | 1.58087 | 0.00590 | 1.58507 | 0.05155 | 1.56360 | 0.02404 | 1.51010 | 0.00217 | 1.48970 | 0.00902 | 1.59103 | 0.03435 |
| 10 | 1.48953 | 0.01560 | 1.44563 | 0.05116 | 1.45100 | 0.04021 | 1.58770 | 0.20726 | 1.41707 | 0.00792 | 1.41530 | 0.02081 | 1.40303 | 0.02945 |
| 1 | 1.42103 | 0.01299 | 1.37520 | 0.05793 | 1.38513 | 0.02782 | 1.41253 | 0.03412 | 1.32767 | 0.00905 | 1.35270 | 0.00581 | 1.38090 | 0.05077 |
| 0 | 1.39620 | 0.00583 | 1.37153 | 0.01371 | 1.37633 | 0.02418 | 1.38677 | 0.02422 | 1.33560 | 0.04761 | 1.28230 | 0.09283 | 1.31237 | 0.03866 |
|  |  |  |  |  |  |  |  |  |  |  |  |  |  |  |
| Fluorescence/ A600 (a.u.) | Average | StDev | Average | StDev | Average | StDev | Average | StDev | Average | StDev | Average | StDev | Average | StDev |
| [Lactate]/ mM | 0TAG | | 1TAG | | 2TAG | | 3TAG | | 10TAG | | 15TAG | | Turbo | |
| 100 | 22568.90 | 1330.46 | 15535.54 | 2731.31 | 13527.80 | 813.40 | 9123.93 | 879.35 | 2536.13 | 386.70 | 1310.71 | 558.73 | 45.23 | 1.56 |
| 10 | 13008.68 | 2113.08 | 8476.91 | 401.36 | 4934.02 | 826.66 | 4570.20 | 1280.85 | 96.46 | 10.24 | 70.62 | 3.67 | 52.74 | 0.33 |
| 1 | 5707.95 | 601.94 | 3275.86 | 361.63 | 2056.14 | 469.13 | 1810.65 | 422.28 | 70.30 | 1.33 | 63.08 | 1.36 | 49.50 | 0.74 |
| 0 (Leak) | 3665.87 | 69.26 | 1214.31 | 148.12 | 708.65 | 83.06 | 569.69 | 101.90 | 50.55 | 20.54 | 67.49 | 3.48 | 49.53 | 0.61 |
|  |  |  |  |  |  |  |  |  |  |  |  |  |  |  |
| Fold-change induction | Average | StDev | Average | StDev | Average | StDev | Average | StDev | Average | StDev | Average | StDev |  |  |
|  | 0TAG | | 1TAG | | 2TAG | | 3TAG | | 10TAG | | 15TAG | |  |  |
|  | 6.15 | 0.27 | 12.75 | 1.05 | 19.20 | 1.73 | 16.61 | 5.01 | 38.46 | 7.42 | 19.30 | 8.02 |  |  |
|  |  |  |  |  |  |  |  |  |  |  |  |  |  |  |
| Readthrough efficiency (%) | Average | StDev | Average | StDev | Average | StDev | Average | StDev | Average | StDev |  |  |  |  |
| [Lactate]/ mM | 1TAG | | 2TAG | | 3TAG | | 10TAG | | 15TAG | |  |  |  |  |
| 100 | 68.84 | 12.10 | 59.94 | 3.60 | 40.43 | 3.90 | 11.24 | 1.71 | 5.81 | 2.48 |  |  |  |  |
| 10 | 65.16 | 3.09 | 37.93 | 6.35 | 35.13 | 9.85 | 0.74 | 0.08 | 0.54 | 0.03 |  |  |  |  |
| 1 | 57.39 | 6.34 | 36.02 | 8.22 | 31.72 | 7.40 | 1.23 | 0.02 | 1.11 | 0.02 |  |  |  |  |
| 0 | 33.12 | 4.04 | 19.33 | 2.27 | 15.54 | 2.78 | 1.38 | 0.56 | 1.84 | 0.09 |  |  |  |  |

**Table S4. Fluorescence readthrough assay data for the leak dampener tool applied to LldR induction using opal (TGA) codons in *E. coli* NEB Turbo**. Data shown in Fig. 4A is tabulated here. For each GFP sample containing the indicated number of TGA codons, fluorescence/A600 values are shown for the average of three biological replicates alongside the corresponding standard deviation values.

| A600 | Average | StDev | Average | StDev | Average | StDev | Average | StDev | Average | StDev |
| --- | --- | --- | --- | --- | --- | --- | --- | --- | --- | --- |
| [Lactate]/ mM | 0TGA | | 1TGA | | 2TGA | | 3TGA | | Turbo | |
| 100 | 1.44367 | 0.07814 | 1.66190 | 0.12944 | 1.67143 | 0.03076 | 1.61570 | 0.11596 | 1.59103 | 0.03435 |
| 10 | 1.05787 | 0.09491 | 1.17360 | 0.01942 | 1.16880 | 0.10907 | 1.17693 | 0.06323 | 1.40303 | 0.02945 |
| 1 | 1.16623 | 0.02008 | 1.17047 | 0.01661 | 1.17850 | 0.11236 | 1.15367 | 0.14091 | 1.38090 | 0.05077 |
| 0 | 1.14897 | 0.04471 | 1.17843 | 0.04666 | 1.13367 | 0.14476 | 1.11793 | 0.07135 | 1.31237 | 0.03866 |
|  |  |  |  |  |  |  |  |  |  |  |
| Fluorescence/ A600 (a.u.) | Average | StDev | Average | StDev | Average | StDev | Average | StDev | Average | StDev |
| [Lactate]/ mM | 0TGA | | 1TGA | | 2TGA | | 3TGA | | Turbo | |
| 100 | 32770.99 | 1220.16 | 9940.22 | 290.33 | 7811.72 | 1376.58 | 3702.25 | 518.77 | 45.23 | 1.56 |
| 10 | 25633.36 | 3737.14 | 1171.19 | 274.68 | 506.47 | 180.85 | 105.03 | 1.25 | 52.74 | 0.33 |
| 1 | 6224.25 | 692.01 | 121.55 | 8.09 | 78.84 | 12.14 | 72.81 | 11.51 | 49.50 | 0.74 |
| 0 (Leak) | 2954.61 | 271.34 | 79.83 | 2.60 | 77.38 | 11.29 | 77.75 | 5.29 | 49.53 | 0.61 |
|  |  |  |  |  |  |  |  |  |  |  |
| Fold-change induction | Average | StDev | Average | StDev | Average | StDev | Average | StDev |  |  |
|  | 0TGA | | 1TGA | | 2TGA | | 3TGA | |  |  |
|  | 11.17 | 1.34 | 124.53 | 1.05 | 100.89 | 10.63 | 47.96 | 9.23 |  |  |
|  |  |  |  |  |  |  |  |  |  |  |
| Readthrough efficiency (%) | Average | StDev | Average | StDev | Average | StDev |  |  |  |  |
| [Lactate]/ mM | 1TGA | | 2TGA | | 3TGA | |  |  |  |  |
| 100 | 30.33 | 0.89 | 23.84 | 4.20 | 11.30 | 1.58 |  |  |  |  |
| 10 | 4.57 | 1.07 | 1.98 | 0.71 | 0.41 | 0.00 |  |  |  |  |
| 1 | 1.95 | 0.13 | 1.27 | 0.19 | 1.17 | 0.18 |  |  |  |  |
| 0 | 2.70 | 0.09 | 2.62 | 0.38 | 2.63 | 0.18 |  |  |  |  |

**Table S5. Fluorescence readthrough assay data for the leak dampener tool applied to LldR regulation of GFP through conditional translation alone in *E. coli* C321.ΔA.exp**. Data shown in Fig. S1A is tabulated here. For each GFP sample containing the indicated number of TAG codons, fluorescence/A600 values are shown for the average of three biological replicates alongside the corresponding standard deviation values.

| A600 | Average | StDev | Average | StDev | Average | StDev | Average | StDev | Average | StDev |
| --- | --- | --- | --- | --- | --- | --- | --- | --- | --- | --- |
| [Lactate]/ mM | 0TAG | | 1TAG | | 2TAG | | 10TAG | | 15TAG | |
| 100 | 1.29727 | 0.20127 | 1.54110 | 0.09106 | 1.43470 | 0.07300 | 0.94383 | 0.04036 | 1.15650 | 0.17704 |
| 10 | 1.40913 | 0.06371 | 1.55070 | 0.10582 | 1.51687 | 0.02513 | 1.17667 | 0.04869 | 1.19253 | 0.00879 |
| 1 | 1.46780 | 0.01642 | 1.39733 | 0.07550 | 1.39290 | 0.08341 | 1.20357 | 0.05960 | 1.16057 | 0.03376 |
| 0 | 1.40430 | 0.08806 | 1.23500 | 0.08969 | 1.25567 | 0.08257 | 1.17190 | 0.03098 | 1.17653 | 0.01805 |
|  |  |  |  |  |  |  |  |  |  |  |
| Fluorescence/ A600 (a.u.) | Average | StDev | Average | StDev | Average | StDev | Average | StDev | Average | StDev |
| [Lactate]/ mM | 0TAG | | 1TAG | | 2TAG | | 10TAG | | 15TAG | |
| 100 | 28902.41 | 4007.96 | 31911.29 | 2255.49 | 32914.34 | 1825.06 | 30872.07 | 867.23 | 31857.91 | 1603.26 |
| 10 | 28274.89 | 2073.47 | 31120.19 | 137.56 | 32714.04 | 729.44 | 26266.02 | 3382.41 | 25120.91 | 3264.73 |
| 1 | 27505.79 | 1528.07 | 29847.41 | 1970.78 | 30079.83 | 628.30 | 21167.81 | 1350.98 | 19839.91 | 1194.07 |
| 0 (Leak) | 27322.67 | 1141.67 | 27477.53 | 1222.41 | 28949.81 | 1049.56 | 20171.22 | 955.44 | 18688.63 | 1831.91 |
|  |  |  |  |  |  |  |  |  |  |  |
| Fold-change induction | Average | StDev | Average | StDev | Average | StDev | Average | StDev | Average | StDev |
|  | 0TAG | | 1TAG | | 2TAG | | 10TAG | | 15TAG | |
|  | 1.06 | 0.11 | 1.16 | 0.05 | 1.14 | 0.04 | 1.53 | 0.12 | 1.71 | 0.12 |
|  |  |  |  |  |  |  |  |  |  |  |
| Readthrough efficiency (%) | Average | StDev | Average | StDev | Average | StDev | Average | StDev |  |  |
| [Lactate]/ mM | 1TAG | | 2TAG | | 10TAG | | 15TAG | |  |  |
| 100 | 110.41 | 7.80 | 113.88 | 6.31 | 106.81 | 3.00 | 110.23 | 5.55 |  |  |
| 10 | 110.06 | 0.49 | 115.70 | 2.58 | 92.90 | 11.96 | 88.85 | 11.55 |  |  |
| 1 | 108.51 | 7.16 | 109.36 | 2.28 | 76.96 | 4.91 | 72.13 | 4.34 |  |  |
| 0 | 100.57 | 4.47 | 105.96 | 3.84 | 73.83 | 3.50 | 68.40 | 6.70 |  |  |

**Table S6. Fluorescence readthrough assay data for the leak dampener tool applied to AraC regulation of GFP containing amber codons in *E. coli* S1030**. Data shown in Fig. S4 is tabulated here. For each GFP sample containing the indicated number of TAG codons, fluorescence/A600 values are shown for the average of two biological replicates alongside the corresponding standard deviation values.

| A600 | Average | StDev | Average | StDev | Average | StDev | Average | StDev | Average | StDev |
| --- | --- | --- | --- | --- | --- | --- | --- | --- | --- | --- |
| [Ara]/ mM | 0TGA | | 1TGA | | 2TGA | | 3TGA | | S1030 | |
| 1 | 1.39730 | 0.12077 | 1.40330 | 0.01570 | 1.42905 | 0.00177 | 1.42140 | 0.001556 | 1.42225 | 0.010819 |
| 0.1 | 1.28125 | 0.04292 | 1.44545 | 0.07403 | 1.44035 | 0.00516 | 1.43355 | 0.006152 | 1.41105 | 0.002616 |
| 0.01 | 1.42525 | 0.00997 | 1.42760 | 0.00863 | 1.42545 | 0.00375 | 1.41510 | 0.005374 | 1.42 | 0.019233 |
| 0.001 | 1.34830 | 0.02079 | 1.38785 | 0.00530 | 1.37025 | 0.00163 | 1.37105 | 0.006859 | 1.43455 | 0.010394 |
| 0.0001 | 1.34200 | 0.00453 | 1.38055 | 0.00134 | 1.37820 | 0.00410 | 1.37725 | 0.011526 | 1.51475 | 0.012374 |
| 0 | 1.33265 | 0.03741 | 1.37945 | 0.00544 | 1.38875 | 0.00092 | 1.37405 | 0.00502 | 1.4217 | 0.010465 |
|  |  |  |  |  |  |  |  |  |  |  |
| Fluorescence/ A600 (a.u.) | Average | StDev | Average | StDev | Average | StDev | Average | StDev | Average | StDev |
| [Ara]/ mM | 0TGA | | 1TGA | | 2TGA | | 3TGA | | S1030 | |
| 1 | 31848.31 | 3066.01 | 29779.63 | 540.62 | 28991.96 | 43.31 | 27973.30 | 323.13 | 46.03 | 6.11 |
| 0.1 | 34899.79 | 2160.32 | 30356.08 | 1356.19 | 29931.17 | 140.65 | 28562.44 | 383.50 | 42.87 | 1.42 |
| 0.01 | 15930.77 | 467.66 | 12111.82 | 271.55 | 12616.92 | 1449.55 | 10366.68 | 39.58 | 44.72 | 0.11 |
| 0.001 | 751.10 | 28.28 | 466.59 | 20.63 | 353.22 | 3.52 | 285.53 | 8.37 | 44.61 | 1.65 |
| 0.0001 | 125.56 | 2.21 | 62.29 | 0.06 | 49.70 | 1.39 | 46.11 | 1.93 | 40.94 | 1.27 |
| 0 | 85.89 | 2.36 | 53.65 | 2.26 | 45.73 | 1.56 | 46.22 | 2.74 | 46.07 | 1.15 |
|  |  |  |  |  |  |  |  |  |  |  |
| Fold-change induction | Average | StDev | Average | StDev | Average | StDev | Average | StDev |  |  |
|  | 0TGA | | 1TGA | | 2TGA | | 3TGA | |  |  |
|  | 370.47 | 25.50 | 555.79 | 33.51 | 634.40 | 20.67 | 606.10 | 28.97 |  |  |
|  |  |  |  |  |  |  |  |  |  |  |
| Readthrough efficiency (%) | Average | StDev | Average | StDev | Average | StDev |  |  |  |  |
| [Ara]/ mM | 1TGA | | 2TGA | | 3TGA | |  |  |  |  |
| 1 | 93.50 | 1.70 | 91.03 | 0.14 | 87.83 | 1.01 |  |  |  |  |
| 0.1 | 86.98 | 3.89 | 85.76 | 0.40 | 81.84 | 1.10 |  |  |  |  |
| 0.01 | 76.03 | 1.70 | 79.20 | 9.10 | 65.07 | 0.25 |  |  |  |  |
| 0.001 | 62.12 | 2.75 | 47.03 | 0.47 | 38.01 | 1.11 |  |  |  |  |
| 0.0001 | 49.61 | 0.05 | 39.58 | 1.11 | 36.73 | 1.53 |  |  |  |  |
| 0 | 62.47 | 2.63 | 53.24 | 1.81 | 53.81 | 3.19 |  |  |  |  |

**Table S7. Mutation rate assay data**. Data shown in Figure 5A is tabulated here. For each sample, mutation rate values shown (µ_bp_, substitutions per base pair per generation) are the average of four biological replicates and their corresponding standard deviation values. For experimental details, see Materials and Methods.

| **Strain #** | **Induction status** | **Strain** | **µBP** | **St Dev** |
| --- | --- | --- | --- | --- |
| 1 | Glucose-repressed | MP6 + JH474 (GLU) | 1.91E-09 | 1.33E-09 |
| 1 | Arabinose-induced | MP6 + JH474 (ARA) | 1.36E-07 | 8.05E-08 |
| 2 | Glucose-repressed | MP7 + JH474 (GLU) | 6.32E-11 | 4.88E-11 |
| 2 | Arabinose-induced | MP7 + JH474 (ARA) | 3.42E-08 | 1.55E-08 |
| 3 | Glucose-repressed | JH474 (GLU) | 4.41E-11 | 3.94E-11 |
| 3 | Arabinose-induced | JH474 (ARA) | 1.44E-10 | 6.68E-11 |
